# Genome-scale quantification and prediction of pathogenic stop codon readthrough by small molecules

**DOI:** 10.1038/s41588-024-01878-5

**Published:** 2024-08-22

**Authors:** Ignasi Toledano, Fran Supek, Ben Lehner

**Affiliations:** 1 Institute for Research in Biomedicine (IRB Barcelona), The Barcelona Institute of Science and Technology (BIST), Barcelona, Spain; 2 Centre for Genomic Regulation (CRG), The Barcelona Institute of Science and Technology (BIST), Barcelona, Spain; 3 Biotech Research and Innovation Centre (BRIC), University of Copenhagen, Copenhagen, Denmark; 4 Institució Catalana de Recerca i Estudis Avançats (ICREA), Barcelona, Spain; 5 University Pompeu Fabra (UPF), Barcelona, Spain; 6 Wellcome Sanger Institute, Wellcome Genome Campus, Hinxton, UK

**Keywords:** Genomics, Clinical trial design, Genetics research

## Abstract

Premature termination codons (PTCs) cause ~10–20% of inherited diseases and are a major mechanism of tumor suppressor gene inactivation in cancer. A general strategy to alleviate the effects of PTCs would be to promote translational readthrough. Nonsense suppression by small molecules has proven effective in diverse disease models, but translation into the clinic is hampered by ineffective readthrough of many PTCs. Here we directly tackle the challenge of defining drug efficacy by quantifying the readthrough of ~5,800 human pathogenic stop codons by eight drugs. We find that different drugs promote the readthrough of complementary subsets of PTCs defined by local sequence context. This allows us to build interpretable models that accurately predict drug-induced readthrough genome-wide, and we validate these models by quantifying endogenous stop codon readthrough. Accurate readthrough quantification and prediction will empower clinical trial design and the development of personalized nonsense suppression therapies.

## Abstract (web summary)

Small molecules can promote translational readthrough of premature termination codons, reducing their pathological effect. This study quantifies the readthrough of ~5,800 human pathogenic stop codons by eight drugs and builds models to predict drug-induced readthrough genome-wide.

## Main

Premature termination codons (PTCs) are the cause of 10%^1^ to 20%^2^ of inherited diseases and an important mechanism of tumor suppressor gene inactivation in cancer. PTCs cause the production of truncated versions of proteins, which are typically loss-of-function and sometimes gain-of-function or dominant negatives. Many, but not all, PTCs also cause the degradation of mRNA transcripts by a process called nonsense-mediated mRNA decay (NMD), strongly reducing the production of the truncated protein^3,4^.

**Fig. 1:**
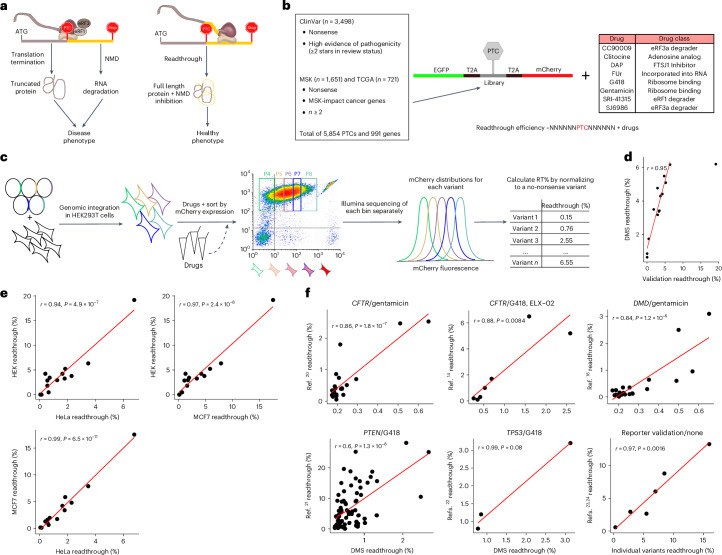
Quantifying readthrough of thousands of pathogenic PTCs. **a**, Readthrough drugs stimulate full-length protein synthesis and decrease NMD-mediated transcript degradation. **b**, Experimental design, ~5,800 nonsense variants in human genetic diseases and cancer were retrieved from ClinVar, TCGA and MSK-IMPACT datasets, cloned in a readthrough reporter, integrated into the genome of HEK293T_LP human cell line and treated with eight readthrough compounds. A readthrough efficiency value was obtained for each variant–drug pair. **c**, Sort-sequencing overview. Each cell integrates one copy of one variant, cells are sorted based on mCherry fluorescence (*x*-axis), bins are sequenced and readthrough percentages are calculated from the mCherry distribution of reads of each variant normalized to the distribution of a no-nonsense variant. **d**, Deep mutational scanning (DMS) versus individual measurements Pearson’s correlation (*r* = 0.95), where 15 variants spanning the whole readthrough range under SRI treatment were individually measured (Spearman correlation (*ρ*) = 0.86). **e**, The same 15 variants shown in **d** were episomally transfected in MCF7 and HeLa cells, and their readthrough percentages were correlated with HEK293T_LP’s. Pearson’s correlation and *P* values are shown. **f**, DMS Pearson’s correlation (and corresponding *P* values) with measurements from previous studies^14,16,20–24^ (Spearman’s correlation (*ρ*) = 0.56, 0.93, 0.71, 0.59, 1, 0.94, from top-left to bottom-right plots). Titles indicate the gene for which nonsense variants were tested and the drug used to stimulate readthrough. The bottom-right plot does not show DMS estimates, but measurements of individual variants also tested in refs. ^23,24^, which were used to validate the readthrough reporter. Note that the readthrough scales differ across some of the studies, illustrating how differences in the assay, conditions and reporter influence the absolute readthrough. Source data

A general therapeutic strategy to alleviate the effects of PTCs would be to promote translational readthrough (RT) of the stop codon (Fig. 1a). Effective nonsense suppression therapy would increase the expression of full-length proteins, reduce the production of pathological protein fragments and inhibit NMD^5^.

Multiple small-molecule drugs that promote PTC-readthrough have been discovered, with diverse mechanisms of action (MOAs) promoting the recognition of stop codons by near-cognate tRNAs rather than translation termination factors^6^. For multiple disease genes, even modest readthrough can be sufficient to alleviate disease symptoms in animal models^7–10^.

The extent of readthrough promoted by small molecules varies extensively for different stop codons, with most drugs increasing the readthrough of UGA more effectively than UAG and UAA PTCs^11–13^. Testing small numbers of mutations has identified sequence features that influence the readthrough of particular stops, for example, the presence of a cytosine in position +1 after the PTC^14^ and the presence of an adenine at position −1 (ref. ^15^). To date, the largest survey of drug-induced readthrough tested the compound TLN468 on 40 variants^16^.

Here we deploy a deep mutational scanning (DMS) approach to generate much richer datasets quantifying nonsense suppression by different drugs. We measure the readthrough of ~5,800 human disease-causing PTCs for eight different readthrough-promoting compounds (henceforth referred to as drugs). We find that the drugs vary substantially in their efficacy and also in the identity of the PTCs that they most effectively promote readthrough. We identify multiple local sequence determinants that predict PTC-readthrough efficacy and show that these determinants differ across drugs. Using these sequence determinants, we are able to build models that predict readthrough efficacy by the best-performing drugs with very good performance genome-wide (*r*^2^ = 0.83). We make these models available as a resource to allow these drugs to be profiled for all possible PTCs in the human genome. Our data and models suggest that the design of clinical trials of nonsense suppression therapies could be improved by using patient–drug combinations that are predicted to be effective.

## Results

### Quantifying readthrough of thousands of pathogenic PTCs

**Extended Data Fig. 1:**
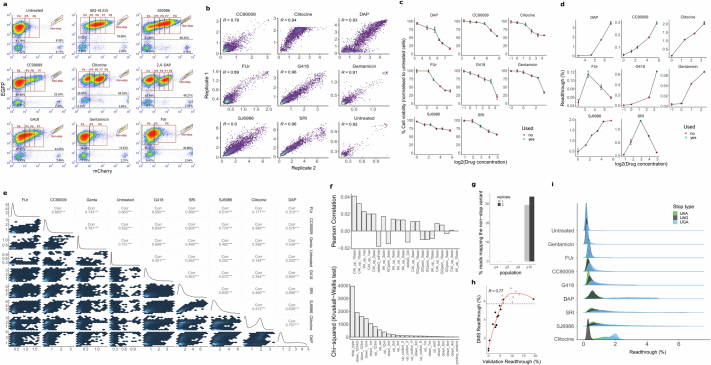
Experimental setup and overview of the drug’s datasets. **a**, FACS profiles (BD Influx Cell Sorter instrument) of the PTC library under the different treatments sorted by EGFP (y-axis) and mCherry (x-axis). Binned populations are indicated together with the control population harboring the no-nonsense *TP53* variant. **b** Inter-replicate correlations for the nine conditions. **c**,**d**, Cell viability (**c**) and readthrough (**d**) titration curves for each drug, where error bars represent the standard deviation across three biological replicates. Four to six drug concentrations were tested for each drug, and the concentration displaying the highest readthrough and reducing cell viability less than 25% (blue) was used for the assay. Very toxic concentrations were not tested for readthrough stimulation. In **d**, readthrough was calculated as the (mCherry^+^ and EGFP^+^)/(EGFP^+^) cell ratio multiplied by the mean mCherry intensity of the mCherry^+^ population and normalized to the readthrough of the no-nonsense variant. **e**, All pairwise inter-drug correlations. **f**, Sequence features association with readthrough efficiency: showing Pearson correlations (continuous variables) and Kruskal–Wallis chi-squared statistics (discrete variables). **g**, Percentage of reads mapping to the no-nonsense variant across sorting populations of the natural stops library under gentamicin treatment. The variant is almost exclusively found in the no-nonsense population, where it represents 30% of the cells. **h**, DMS vs individual measurements Pearson’s correlation (r = 0.77). It represents an extension of Fig. 1d to specifically test the upper ceiling of the assay, with ten more variants spanning high-readthrough estimates included. A loess curve was fit to model the non-linearities triggered by the upper limit of the assay (see Supplementary Note 1 for more information on the assay saturation limit. Variants above the dashed line (1.3% of the library) have >90% reads in the highest sorting gate. **i**, Readthrough distributions across drugs colored by the stop type. Source data

To quantify drug-induced readthrough of diverse PTCs, we constructed a library containing 3,498 PTCs that cause Mendelian diseases reported in ClinVar^1^, 2,372 recurrent somatic PTCs in cancer genes (721 from The Cancer Genome Atlas (TCGA)^17^ plus 1,651 from MSK-IMPACT^18^) and a *TP53* control no-nonsense variant (*n* = 5,871; Fig. 1b; Methods). We cloned each PTC with 144 nucleotides (nts) of surrounding sequence context into a dual fluorescent protein reporter, where an upstream green fluorescent protein (EGFP) controls for variable expression and readthrough causes expression of a downstream mCherry protein, and performed single-copy genomic integration into HEK293T landing pad (LP) cell line^19^. We combined fluorescence sorting and Illumina sequencing to obtain readthrough efficiencies (Fig. 1c and Extended Data Fig. 1a), which were highly correlated across replicates (Extended Data Fig. 1b), with individual measurements of 15 variants spanning the full dynamic range of the assay in HEK293T_LP cells (*r* = 0.95, *ρ* = 0.86; up to the measurement saturation limit of ~6%; Fig. 1d, Extended Data Fig. 1h, Supplementary Table 1 and Supplementary Note 1) and in two other cell lines (MCF7 and HeLa; Fig. 1e). Readthrough is highly correlated across variants in the three cell types (*r* = 0.94–0.99), but absolute readthrough levels are about twofold lower in HeLa cells. Our measurements also correlate very well with quantifications performed in other laboratories comprising varied genes and drugs^14,16,20–24^ (Fig. 1f).

### Readthrough varies extensively across drugs and PTCs

**Fig. 2:**
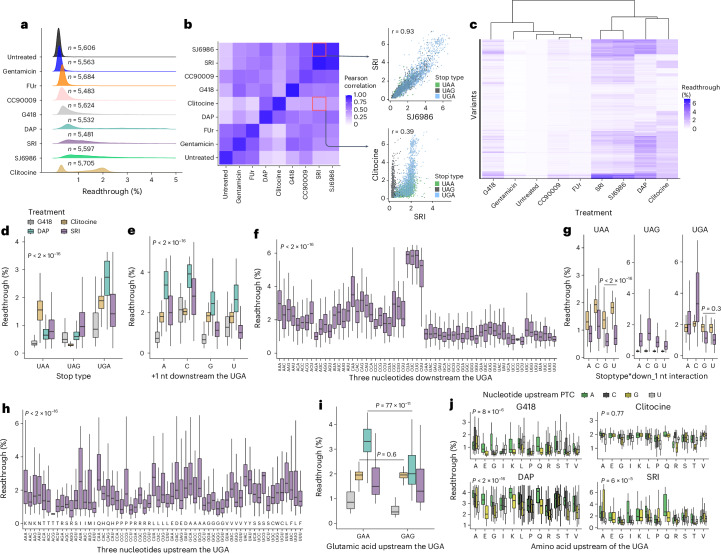
Sequence features explain the readthrough variability across PTCs and drugs. **a**, Readthrough distributions across drugs. The number of high-confidence variants (≥10 reads) recovered for each treatment and for which readthrough percentages were quantified is shown. **b**, Interdrug correlation. Correlation values between the same drug represent the inter-replicate correlation. Examples of high-correlated (SRI and SJ6986) and low-correlated (SRI and clitocine) drug pairs are shown, colored by stop type. **c**, Readthrough efficiencies for all variant–drug combinations. **d**–**j**, Effect of the sequence feature (*x* axis) on readthrough efficiency (*y* axis) in HEK293T_LP cells, colored by the drug. The top and bottom sides of the box are the upper and lower quartiles, respectively. The box covers the interquartile interval, where 50% of the data are found. The horizontal line that splits the box in two is the median. Only variants where the stop codon is UGA are shown (except for **d** and **g**, where all stop codon variants are shown). The sequence features are stop codon identity (*n* = 22,342, *P* < 2 × 10^−16^, Kruskal–Wallis test; **d**), the nucleotide in position +1 downstream of the PTC (*n* = 10,602, *P* < 2 × 10^−16^; **e**), the nucleotides in +1, +2 and +3 positions downstream of the PTC (*n* = 2,589, *P* < 2 × 10^−16^; **f**), same as **e** but stratified by stop codon (in clitocine samples U>G for UAA stops, *n* = 614, adjusted *P* < 2 × 10^−16^; U=G for UGA stops, *n* = 1,395, adjusted *P* = 0.3; one-sided Wilcoxon signed-rank test; **g**), the nucleotides in −1, −2 and −3 positions upstream of the PTC together with the amino acid encoded by each codon (*n* = 2,589, *P* < 2 × 10^−^^16^, Kruskal–Wallis test; **h**) and same as **h** but only for variants with a glutamic acid upstream of the PTC (GAA>GAG for DAP, *n* = 155, adjusted *P* = 7 × 10^−11^; GAA=GAG for clitocine, *n* = 158, adjusted *P* = 0.6; one-sided Wilcoxon signed-rank test; **i**). Finally, the effect of amino acids encoded by A-ending codons on readthrough efficiency across drugs, where codons ended in A display higher readthrough compared to the rest of the codons (*n* = 7,989, adjusted *P* < 6 × 10^−5^ for DAP, G418 and SRI, one-sided Wilcoxon signed-rank test). The nucleotide upstream of the PTC is colored (**j**). Source data

**Extended Data Fig. 2:**
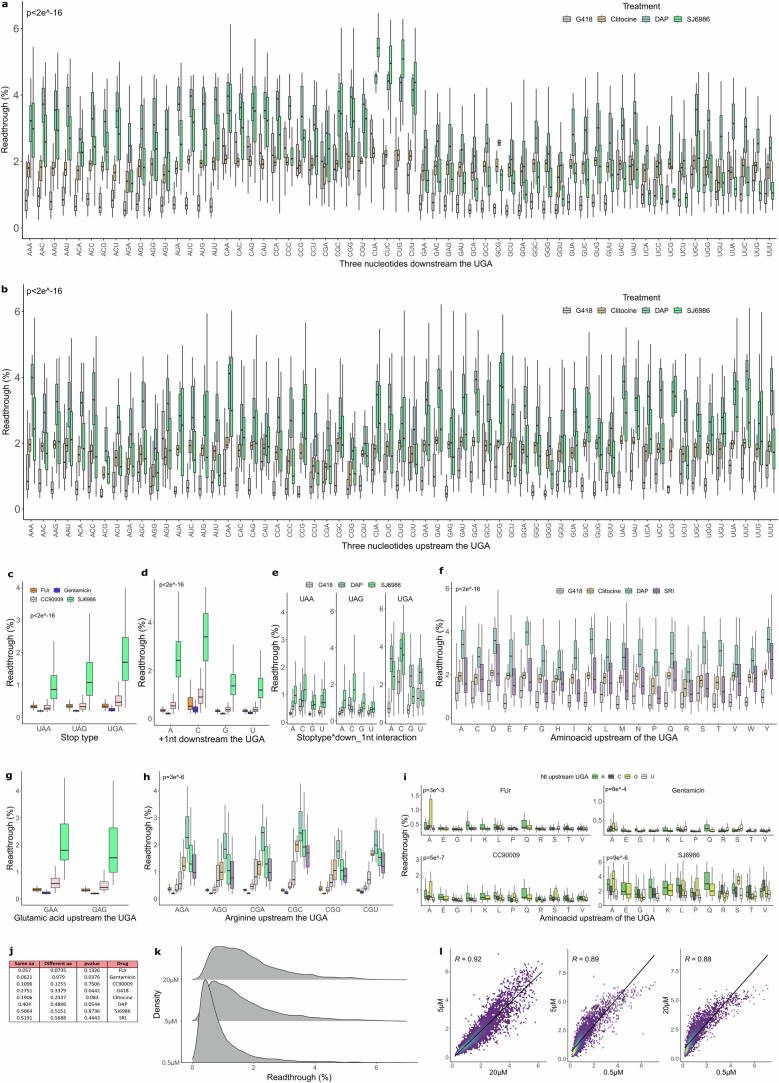
Sequence features explain the readthrough variability across PTCs and drugs. **a**–**i**, Effect of the sequence feature (*x*-axis) on readthrough efficiency (*y*-axis) colored by drug. The top and bottom sides of the box are the lower and upper quartiles. The box covers the interquartile interval, where 50% of the data are found. The horizontal line that splits the box in two is the median. Only variants where the stop codon is UGA are shown (except for **c** and **e**, where all stop codon variants are shown). The sequence features are the three nucleotides downstream of the PTC (n = 10645, p < 2e−16, Kruskal–Wallis test) (**a**), the three nucleotides upstream of the PTC (n = 10645, p < 2e−16) (**b**), the stop type (n = 22227, p < 2e−16) (**c**), the nucleotide in position +1 downstream of the PTC (n = 10502, p < 2e−16) (**d**), same as **d** but stratified by stop codon (n = 16753) (**e**), the amino acid upstream of the PTC (n = 10602, p < 2e−16) (**f**), variants with a glutamic acid upstream of the PTC stratified by the codon (n = 613) (**g**), variants with an arginine upstream of the PTC stratified by the codon (n = 1040, p = 3e−6) (**h**), and the effect of amino acids encoded by A-ending codons on readthrough efficiency for FUr, gentamicin, CC90009 and SJ6986, where codons ended in A display higher readthrough efficiencies compared to the rest of the codons (n = 7902, adjusted p < 1e−3, one-sided Wilcoxon signed-rank test). The nucleotide in position +3 of the codon is denoted with colors (**i**). **j**, Mean readthrough difference between pairs of codons with Hamming distance of 1 (that is, single nucleotide difference) that encode for the same amino acid, or pairs that encode a different amino acid across drugs. *P* value of the two-sided t-test between the same amino acid and different amino acid groups is shown. **k**,**l**, Readthrough distributions (**k**) and pairwise correlations (**l**) for the three SJ6986 concentrations tested (0.5 μM, 5 μM and 20 μM). Source data

We tested four to six concentrations for 20 drugs reported to induce readthrough, of which eight induced reproducible readthrough in our assay (Extended Data Fig. 1c,d and Supplementary Table 2; Methods). We quantified the readthrough of the library in untreated conditions and under the effect of the following eight drugs: CC90009 (refs. ^25,26^), clitocine^27^, 2,6-diaminopurine (DAP)^12^, gentamicin^28,29^, G418 (refs. ^28,29^), SJ6986 (ref. ^25^), SRI-41315 (refs. ^30,31^; henceforth: SRI) and 5-fluorouridine (FUr)^8^, which comprise different classes of small molecules spanning different MOAs (Fig. 1b, Methods). Considering all PTCs in the library, the median readthrough varied across drugs from 0.08% (gentamicin) to 1.32% (SJ6986; Supplementary Table 3). However, each drug promoted a stronger readthrough of a subset of PTCs, with the median readthrough of the top 10% of variants varying from 0.51% (gentamicin) to 4.28% (DAP). Readthrough distributions were unimodal with a long upper tail for seven drugs, whereas clitocine treatment resulted in a bimodal distribution (Fig. 2a and Extended Data Fig. 1i). In the absence of drugs, only a very small number of PTCs (*n* = 17) gave >1% readthrough. Additionally, by quantifying readthrough for three SJ6986 concentrations (0.5 μM, 5 μM and 20 μM), we observed that sequence effects are preserved across drug concentrations (Extended Data Fig. 2k,l and Supplementary Note 2).

The readthrough profiles of the different drugs are, in most cases, only moderately correlated (Fig. 2b and Extended Data Fig. 1e). One exception is SRI and SJ6986, which both inhibit eRF1/eRF3 (refs. ^25,30^) and induce readthrough of a highly correlated set of PTCs (*r* = 0.93; Fig. 2b and Supplementary Note 3). The effects of other drugs are much more distinct. Clitocine and SRI, for example, both elicit high readthrough of many PTCs, but their effects are only weakly correlated (*r* = 0.39, in comparison to the inter-replicate correlations of *r* = 0.94 and *r* = 0.96 for the two drugs). Hierarchical clustering of the readthrough profiles of all 5,837 PTCs identifies sets of PTCs with strong readthrough induced by multiple drugs as well as PTCs strongly affected by only one drug (Fig. 2c).

### Stop type and downstream sequence modulate readthrough

To better understand why the readthrough of particular PTCs is promoted by particular drugs, we quantified the association between readthrough and 47 sequence features for all drugs. These included the stop codon type, the adjacent downstream and upstream nucleotides (up to eight nucleotides away), several codon-related metrics and general features such as G + C content and RNA secondary structure propensity (Extended Data Fig. 1f and Supplementary Table 4).

Figure 2 shows data for one drug representative of each MOA, with the remaining drugs presented in Extended Data Fig. 2. Consistent with previous observations^23,32,33^, drug-induced readthrough is much stronger for particular types of stop codon (*P* < 2 × 10^−16^, Kruskal–Wallis test). However, this varies extensively across drugs. For example, for G418 and SRI, the efficiency of readthrough is UGA>UAG>UAA, whereas for clitocine it is UGA>UAA>>UAG, and for DAP, it is UGA>>UAG~UAA (*P* < 1.7 × 10^−15^ for all comparisons, one-sided Wilcoxon signed-rank test; Fig. 2d and Extended Data Fig. 2c). Drugs with the same direction of effect can also have different magnitudes of effects. For instance, both DAP and SRI stimulate UGA>UAG, but the fold change is different (4.65-fold and 1.64-fold, respectively).

To control for the strong effect of the stop codon types, in the following sections, we focus on UGA variants because they trigger the highest readthrough across all drugs (conclusions for UAG and UAA are similar, and all data are included in Supplementary Table 3, with main differences pointed out in the text). The three nucleotides immediately after a stop codon have been previously reported to modulate readthrough efficiency in the absence of drugs^23,34^. Consistent with this, we see a strong effect of the downstream sequence (+1, +2 and +3 nts) on drug-induced readthrough (*P* < 2 × 10^−16^, Kruskal–Wallis test). However, as for stop codon preferences, how the downstream sequence modulates readthrough is drug-specific. Readthrough by all drugs is modulated by the nucleotide immediately after the stop codon (Fig. 2e and Extended Data Fig. 2d), with C consistently being the most efficient nucleotide. However, the rest of the nucleotides show distinct preferences across drugs.

Readthrough is also modulated by the +2 and +3 positions, and the effects differ across drugs (Fig. 2f and Extended Data Fig. 2a). We identified a stop codon-dependent effect of the downstream nucleotides (Fig. 2g and Extended Data Fig. 2e), indicating genetic interactions between neighboring nucleotides. A detailed analysis of nucleotide contexts can be found in Supplementary Note 4.

### Upstream sequence modulates readthrough

Previous studies in bacteria^35^, yeast^36,37^ and mammalian cells^38,39^ have shown that the codons upstream of a stop codon can also modulate readthrough under drug-free conditions. Clustering sequences in our library by the upstream codon revealed upstream preferences for each of the drugs (*P* < 2 × 10^−16^, Kruskal–Wallis test; Fig. 2h and Extended Data Fig. 2b). For instance, under SRI treatment, the codons encoding the amino acids P, G and I (*n* = 193) display low readthrough, as opposed to Y- and Q-encoding codons (*n* = 459), which drive high readthrough (1.8-fold in Q and Y versus P, G and I; *P* < 2 × 10^−16^, one-sided Wilcoxon signed-rank test; Extended Data Fig. 2f). Note that codons encoding the same amino acid might display different readthrough in a drug-specific fashion (Fig. 2i and Extended Data Fig. 2g,h).

To gain more insight into the effect of the upstream sequence, we clustered the codons by the identity of the third nucleotide (Fig. 2j and Extended Data Fig. 2i). Codons ending in A (*n* = 538 versus *n* = 1,457) tend to be the top-readthrough-promoting codons for all readthrough drugs, except for clitocine, although the effect differs across amino acids (1.1- to 1.3-fold change, adjusted *P* < 1 × 10^−4^ for DAP, G418, SRI, SJ6986 and CC90009). Additional analyses of nucleotide contexts can be found in Supplementary Note 4.

**Extended Data Fig. 3:**
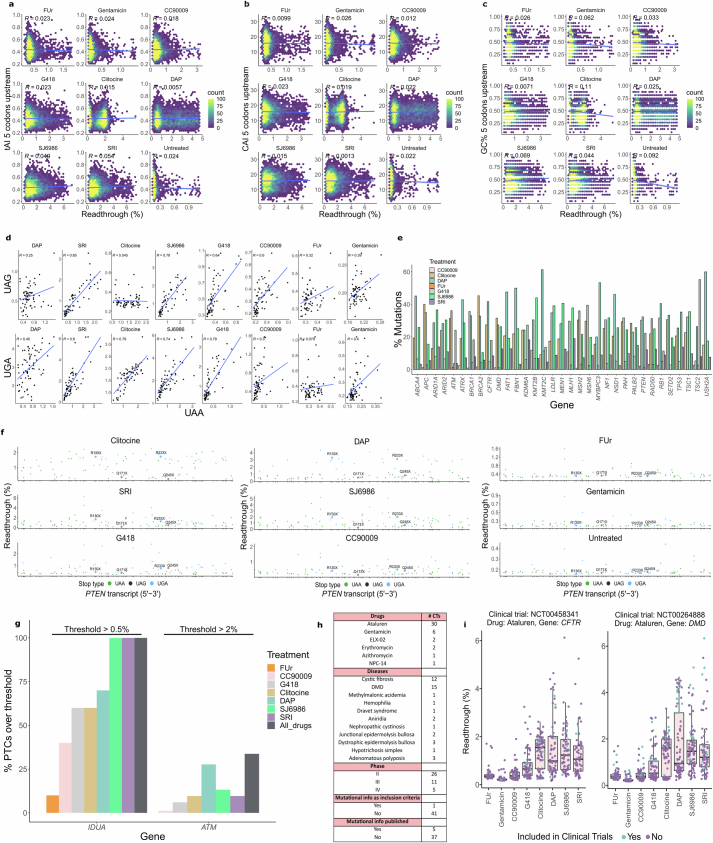
Codon-related features, multistop variants and overview of PTEN nonsense mutations and clinical trials. **a**–**c**, Correlation of tAI (**a**), CAI (**b**) and GC% (**c**) of the 5aas upstream of the PTC with readthrough efficiency for each drug. **d**, Correlation of multistop variants across drugs. Each data point belongs to a mutation in the same genomic position but with a different stop type. **e**, Drug preferences for the highly represented genes in our dataset (>20 variants, n = 33). Y-axis shows the percentage of mutations for which each drug displays the highest readthrough. **f**, Readthrough efficiency across drugs for 97 nonsense *PTEN* mutations colored by stop codon type. The top 4 most recurrent nonsense mutations in human tumors are highlighted. **g**, Percentage of *IDUA* and *ATM* mutations in our dataset displaying higher readthrough levels than the phenotypic threshold reported in refs. ^7,43,44^ across drugs. **h**, All past and current (n = 42) phases II–IV clinical trials testing readthrough drugs, obtained from ref. ^45^. **i**, Our readthrough efficiencies of the nonsense variants tested in two clinical trials (CTs) (blue), together with the rest of nonsense variants in the same gene tested in our assay (purple). Clinical trial identifier, drug (ataluren) and gene tested are specified in the titles. Many variants included in clinical trials are unresponsive to drugs, likely hindering their performance. The top and bottom sides of the box are the lower and upper quartiles. The box covers the interquartile interval, where 50% of the data are found. The horizontal line that splits the box in two is the median. Source data

We found little association between readthrough and GC-content or codon bias indexes (codon adaptation index (CAI)^40^, tRNA adaptation index (tAI)^41,42^; Methods; Extended Data Fig. 3a–c). Controlling for nucleotide sequence does suggest an additional effect of the encoded amino acid (Extended Data Fig. 2j).

### Multistop variants

**Fig. 3:**
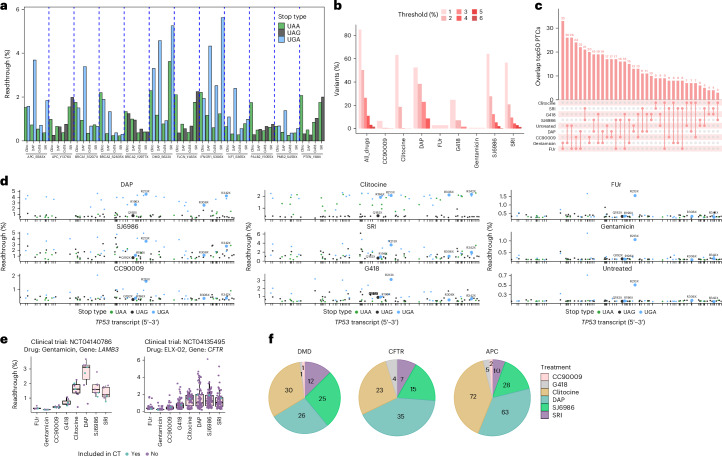
Readthrough-sensitive nonsense variants differ across drugs. **a**, Readthrough efficiency for 12 multistop variants across four drugs. Each multistop variant comprises two different nonsense mutations (different stop codon identities) observed in the same genomic locus. **b**, Percentage of variants with readthrough over different thresholds for each drug separately and when considering all eight drugs together (All_drugs). **c**, All pairwise overlaps of each drug’s top 50 readthrough-sensitive variants. The number indicates how many variants overlap in the top 50 readthrough-sensitive variant sets of the two compared drugs. **d**, Readthrough efficiency across drugs, for 102 nonsense *TP53* mutations colored by stop codon type. The top five most recurrent nonsense mutations in the human tumor genomes are highlighted. **e**, Our observed readthrough efficiencies of the nonsense variants tested in two clinical trials (CTs) (blue), together with the rest of the nonsense variants in the same gene tested in our assay (purple). Clinical trial identifier, drug and gene tested are specified in the titles. The top and bottom sides of the box are the lower and upper quartiles. The box covers the interquartile interval, where 50% of the data are found. The horizontal line that splits the box in two is the median. **f**, Number of variants for which each drug displays the highest readthrough efficiency across the top three genes commonly tested in clinical trials for nonsense suppression therapies, considering all variants in our dataset. Source data

Our library comprised a total of 240 genomic positions with two variants representing different stop types (named ‘multistop variants’). The correlation of readthrough between pairs of stop variants ranges from ~0 to almost 0.85, depending on the drug and stop types being compared (Extended Data Fig. 3d). For instance, in SRI and SJ6986, the UGA variants correlate well in readthrough efficiency with the UAA variants, but their readthrough is two times higher. For G418, UGA variants are three times more readthrough sensitive than UAA variants. Other comparisons show very different behavior across stop types (for example, UAA versus UAG for DAP and clitocine). Examples of different stop codon variants in the same genomic position under different treatments are shown in Fig. 3a. For instance, *DMD*_S622X_UGA responds efficiently to DAP, but *DMD*_S622X_UAA responds poorly. Other examples include *PTEN*_Y88X in clitocine, *IFNGR1*_S306X in DAP/G418/SRI and *APC*_S583X in DAP/SRI.

### Effective readthrough drugs for pathogenic variants

The drug-specific readthrough of different variants increases the number of patients potentially treatable by a genetically informed choice of drug. Considering all 5,837 PTCs in our library, readthrough >2% can be achieved for 50.3% by using the best drug for each mutation. This is higher than for any individual drug, with >2% readthrough for 38%, 28%, 21%, 19%, 7% and 0.6% of PTCs with DAP, SJ6986, SRI, clitocine, G418 and CC90009, respectively (Fig. 3b). By applying genetically informed drug selection, many variants display even higher readthrough—>3% for 27% of PTCs, >4% for 11%, >5% for 3.2% and >6% for 1.6% (Fig. 3b).

However, clinical trials usually test one drug—one gene association; hence, knowing which drug maximizes readthrough across all observed PTCs of a gene is important. The highly represented genes in our library (>20 mutations, *n* = 33) revealed that DAP, SJ6986 and clitocine are the most efficient drugs for most genes, but their relative order is different (Extended Data Fig. 3e). As expected, it is strongly associated with the stop type prevalence in the gene. In general, clitocine emerges as the best drug for UAA-rich genes (*APC* and *BRCA2*), DAP for UGA-rich (*ATRX* and *FAT1*) and SJ6986 for UAG-rich (*MYBPC3* and *TSC2*). Additionally, we asked whether some genes are overall more readthrough sensitive than others. The average readthrough when pooling all drugs together is normally distributed and varies between 0.9% and 1.5% across genes (>20 mutations in our library, *n* = 33).

### Readthrough of tumor suppressor and disease genes

As evidenced above, there is considerable drug-specific variability in readthrough at the variant level (Fig. 2c). Comparing the top 50 most sensitive variants for each drug shows that each drug maximizes readthrough of a different set of variants (an average of 13 variants overlap across all pairwise comparisons; Fig. 3c). Thus, the best drug to apply would strongly depend on the particular nonsense mutation causing the disease in each patient.

As an example of how different drugs promote the readthrough of different PTCs, we consider the commonly mutated tumor suppressors *TP53* and *PTEN* (Fig. 3d, Extended Data Fig. 3f and Supplementary Table 5). The five most recurrent *TP53* PTCs constitute 44% of all the *TP53* nonsense mutations in the MSK-IMPACT and TCGA datasets (102 different *TP53* nonsense mutations) and are carried by 3% of all MSK-IMPACT and TCGA patients^17,18^. These PTCs show promising readthrough therapy potential. Readthrough of the most prevalent *TP53* nonsense mutation R213X_UGA can be substantial—4.5% with DAP and 3.6% with SRI and SJ6986. Interestingly, readthrough stimulation of *TP53*_R213X_UGA bearing mice was reported to decrease tumor growth^8^. Readthrough of the second most frequent PTC, R342X_UGA, is strong with clitocine, DAP, SRI and SJ6986 (all > 2%). Q192X_UAG, the fifth most recurrent mutation, is the only variant in this set insensitive to all treatments (readthrough < 1%). In total, readthrough >2% can be achieved by at least one drug for 43/102 PTCs in *TP53*. Considering all 102 PTCs, SJ6986 is the most effective drug for 36 PTCs, DAP for 25 PTCs, clitocine for 21 PTCs, SRI for 14 PTCs and G418 for 6 PTCs in *TP53*. Encouragingly, clitocine treatment was shown to impede tumor growth in mice bearing the Q136X_UAA mutation^27^, which displays a 2.1% readthrough in our dataset. R196X_UGA, R213X_UGA, R306X_UGA and R342X_UGA have similar readthrough efficiencies (1.9%, 2.1%, 2.1% and 2.1%, respectively), suggesting that clitocine could also have similar therapeutic potential for these four *TP53* mutations.

For *PTEN*, the four most frequent PTCs constitute 36% of PTCs reported in MSK-IMPACT (97 different *PTEN* nonsense mutations were considered here). Of these, the two more prevalent mutations (R130X_UGA and R233X_UGA) show very similar readthrough profiles with high DAP-induced readthrough (3.4% and 3.1%) and are also effectively stimulated by SJ6986 (2.0% and 2.0%), clitocine (1.7% and 1.8%) and SRI (1.8% and 1.3%; Extended Data Fig. 3f), in line with previous observations that these mutations drive restoration of functional *PTEN* under readthrough treatment^21^. In contrast, *PTEN* Q171X_UAG and Q245X_UAG do not respond to any of the readthrough drugs tested here (readthrough <1%). In total, readthrough >2% can be achieved for 35/97 of all the PTCs reported in *PTEN* in MSK-IMPACT with at least one drug. Considering all 97 PTCs, DAP is the most effective drug for 32 PTCs, clitocine for 31 PTCs, SJ6986 for 22 PTCs and SRI for 12 PTCs.

Residual WT protein levels that partially rescue phenotypes were also reported for mucopolysaccharidosis type I-Hurler disease (>0.5% expression of *IDUA* gene)^7^ and ataxia-telangiectasia (2–5% expression of *ATM* gene)^43,44^. Our dataset shows that 10/10 *IDUA* mutations and 28/83 *ATM* mutations display readthrough >0.5% and >2%, respectively, for at least one treatment, suggesting that patients harboring those mutations could be promising candidates for readthrough therapy (Extended Data Fig. 3g).

### Genetics-informed patient stratification for clinical trials

Our data highlight the highly variable efficacy of readthrough-inducing drugs across different PTCs. However, to our knowledge, only 1 of 42 phase II–IV clinical trials using readthrough-promoting drugs^45^ used the genetic context of a PTC as an inclusion criterion (ClinicalTrials.gov registration: NCT04135495; Supplementary Table 6 and Extended Data Fig. 3h). Furthermore, only five trials made the identity of patient PTCs available^46–48^.

We used our data to evaluate the optimal match between drugs and PTCs in two of these trials (Fig. 3e). Clinical trials NCT04140786 used gentamicin and NCT04135495 used a gentamicin derivative, ELX-02. However, our data show that effective readthrough of the PTCs present in patients included in these trials is likely to have been very limited. The average readthrough of these PTCs by gentamicin is only 0.2%. In contrast, average readthrough by clitocine and DAP would be 1.8% and 2.9%, respectively, and other PTCs in the same gene would be better choices for a gentamicin trial (Fig. 3e). In the two other trials with available patient data, the most effective drug also varies across patients' PTCs and PTCs display high-readthrough variability within each drug, too (Extended Data Fig. 3i). Patient response data shows a non-significant but positive correlation with our readthrough measurements (Supplementary Note 5).

We next considered the following three genes most frequently targeted in nonsense suppression clinical trials: *DMD*, *CFTR* and *APC*. Our data shows that the most effective readthrough drugs for these genes are clitocine and DAP, but that, in all three cases, a combination of drugs matched to patient PTCs would prompt the highest readthrough rates (Fig. 3f). For example, of the 95 pathogenic PTCs in *DMD*, the most effective readthrough is obtained with clitocine for 30 PTCs, DAP for 26, SJ6986 for 25, SRI for 12 and G418 and CC90009 for 1 PTC each. To our knowledge, none of the best three drugs (clitocine, DAP and SJ6986) have yet been evaluated in clinical trials.

### Accurate prediction of readthrough efficiency

**Extended Data Fig. 4:**
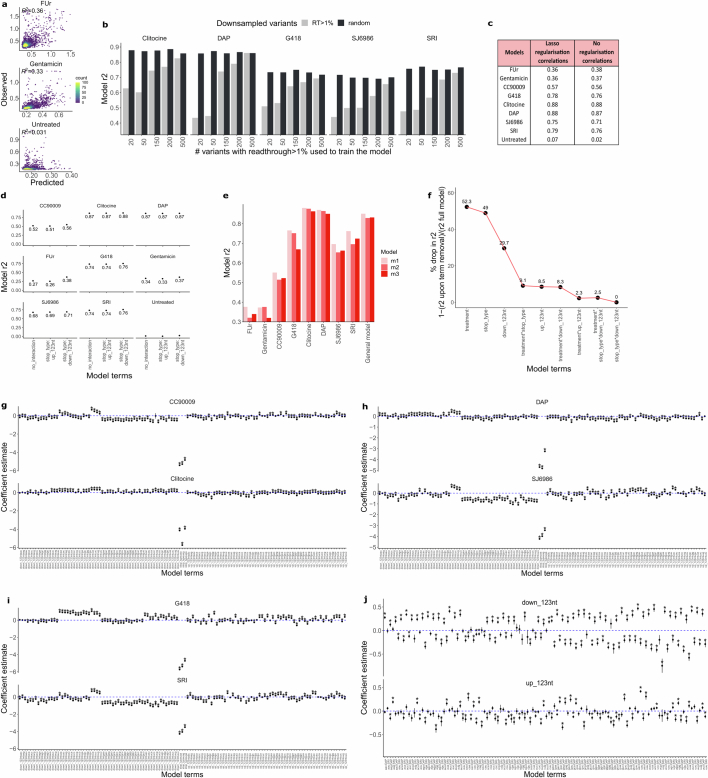
Predictive models overview and optimization. **a**, Drug-specific models cross-validated predictive performance for FUr, gentamicin and untreated conditions. **b**, Downsampling the number of readthrough variants yields decreased model performance for the high-performing drug models (Supplementary Note 6). *X*-axis shows the number of variants with readthrough >1% retained and used to rerun the models (gray). We used control models randomly removing the same number of variants to control for the effect of smaller training sizes in model performance (black). Models with 20, 50 and 150 variants retained intend to represent similar scenarios to untreated, gentamicin and FUr datasets. The r^2^s shown are the average over 10 cross-validation rounds. **c**, Comparison of the r^2^ values for the drug-specific models when using stop type, the three nucleotides downstream and upstream of the PTC and the interaction between stop type and three nucleotides downstream versus when using ElasticNet regularization on 47 sequence features (Extended Data Fig. 1f and Supplementary Table 4). **d**, Performance for three different model formulations across drugs (Supplementary Note 6): using only stop type and the three nucleotides downstream and upstream of the PTC, adding the stop type and three nucleotides upstream interaction or adding the stop type and three nucleotides downstream interaction. Only the latter consistently improves model performance across drugs. The r^2^s shown are the average over 10 cross-validation rounds. **e**, Performance for three different model formulations across drugs: encoding the three nucleotides downstream and upstream of the PTC as nucleotide triplets (m1), encoding the upstream sequence as a nucleotide triplet and the three nucleotides downstream as three different terms (one for each position, no interaction among them, m2) and encoding the downstream sequence as a nucleotide triplet and the three nucleotides upstream as three different terms (one for each position, no interaction among them, m3). m1 consistently yields higher r^2^ across drugs. The r^2^s shown are the average over 10 cross-validation rounds. **f**, Contribution of each sequence feature to the pan-drug model. The y-axis shows the relative drop in r^2^ when each term is removed from the model and normalized to the full model (1 − (r^2^ upon term removal/r^2^ full model)). **g**–**j**, Model coefficients of the following predictive models: CC90009 and clitocine (**g**), DAP and SJ6986 (**h**), G418 and SRI (**i**) and the down_123 nt (top) and up_123 nt (bottom) coefficients for the pan-drug model (**j**). Mean, 95% confidence intervals and significance (two-sided Student’s t-test) of the coefficient estimates across 10 cross-validation rounds are shown. Asterisks represent an adjusted p-value < 0.01. Source data

Our extensive and quantitative dataset of drug-induced PTC readthroughs provides an opportunity to train and evaluate computational models to predict drug-induced readthrough. We focused on the six drugs that triggered readthrough >1% for >3% of PTCs and used logistic regression to train sequence-based genotype–phenotype models (Supplementary Note 6). Models for the remaining three conditions (FUr, gentamicin and untreated cells) had poor predictive performance (*r*^2^ = 0.37, 0.38 and 0.02, respectively) due to the very small proportion of PTCs undergoing any notable amount of readthrough in these conditions (153, 31 and 17 PTCs with >1% readthrough, respectively; Extended Data Fig. 4a,b and Supplementary Note 6).

**Fig. 4:**
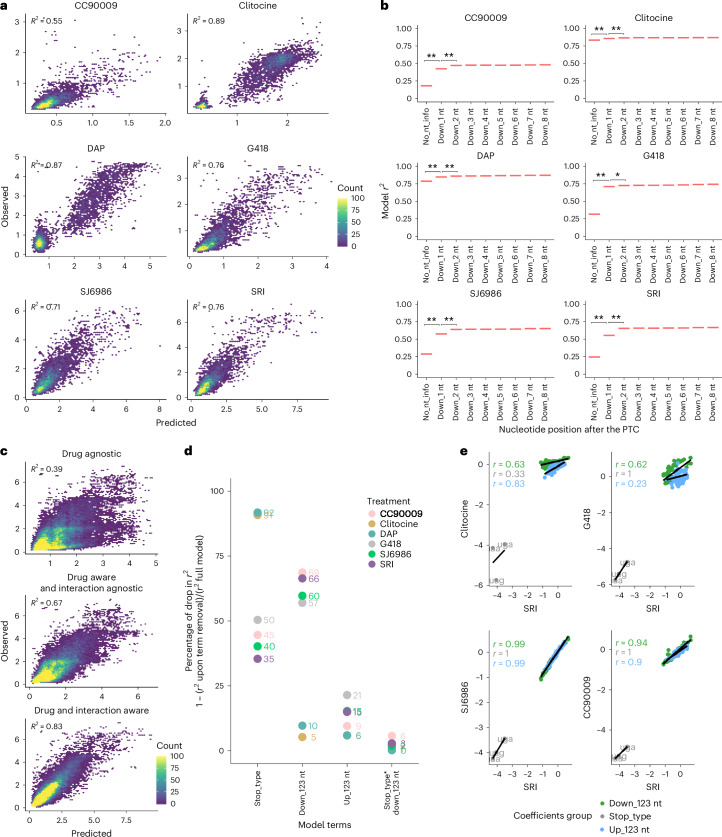
Interpretable models predict readthrough efficiency from sequence context. **a**, Drug-specific models cross-validated predictive performance for CC90009, clitocine, DAP, G418, SJ6986 and SRI conditions. **b**, Contribution to model performance of the eight nucleotides downstream of the PTC (by adding one at a time). The fixed predictive variables present in all models are the stop codon type and the three nucleotides upstream of the PTC. *T* test over 20 cross-validation rounds comparing each model (column) to the previous one was used to determine significance (adjusted *P < 0.05, adjusted **P < 0.01, one-sided *t* test). **c**, Pan-drug models cross-validated predictive performance—drug-agnostic (top), drug-aware but sequence × drug interaction-agnostic (middle), drug and sequence × drug interaction-aware (bottom). **d**, Contribution of each sequence feature to the drug-specific models. *Y* axis shows the percentage drop in *r*^2^ when each term is removed from the model and normalized to the full model (1 − (*r*^2^ on term removal/*r*^2^ full model)). **e**, Correlation of drug-specific model coefficients (note that for the sake of coefficient interpretability, we ran the models without the interaction term stop_type × down_123 nts, which incurs only a small decrease of *r*^2^, ranging between 1% and 3% depending on the drug). Coefficients are colored by the model feature they belong to—stop codon type, down_123 nt and up_123 nt. Drugs displaying high correlations respond similarly to the sequence features and, consequently, trigger readthrough of similar subsets of PTCs. Source data

After model optimization (Supplementary Note 6 and Extended Data Fig. 4c–e), we found that a simple model using four sequence feature groups showed good performance across all six drugs (Fig. 4a and Supplementary Table 7). The four feature groups included are as follows: (1) stop codon type; (2) the three nucleotides downstream of the PTC and their interactions; (3) the three nucleotides upstream of the PTC and their interactions and (4) the interaction between the stop type and the three nucleotides downstream of the PTC (formula 4 in Supplementary Note 8). Increasing the downstream sequence context up to +8 nts didn’t improve predictive performance for any of the drugs (Fig. 4b and formula 5 in Supplementary Note 8). The correlation between predicted and observed readthrough evaluated by ten rounds of cross-validation (90–10% training-testing split) was *r*^2^ = 0.89 (clitocine), 0.87 (DAP), 0.76 (SRI), 0.76 (G418), 0.71 (SJ6986) and 0.55 (CC90009). Of note, CC90009 is the dataset with the highest technical noise (*r*^2^= 0.62 inter-replicate correlation), likely hindering model performance. Training a single model with the data from all six drugs (pan-drug model) provided poor performance (*r*^2^ = 0.39) unless the drug identity was included as a predictive feature with interaction terms between the drug and the additional features (*r*^2^ = 0.83 for the pan-drug model; Fig. 4c, Supplementary Table 8 and formula 6 in Supplementary Note 8). This model explains 94% of the explainable readthrough variance (maximum achievable *r*^2^ = 0.89 calculated via inter-replicate correlation across all variants and drugs).

To identify features important for model performance, we removed one variable at a time and calculated the drop in cross-validated *r*^2^ normalized to the full model *r*^2^, showing that feature contributions quantitatively differ across drug models (Fig. 4d, Extended Data Fig. 4f and Supplementary Note 7). We also compared the model coefficients for each feature (in a simplified model without the interaction term to aid coefficient interpretability; Fig. 4e, Extended Data Fig. 4g–j, Supplementary Note 6 and Supplementary Tables 9 and 10), which allowed us to capture similarities and differences of individual sequence elements across drugs.

### Readthrough prediction for all PTCs in the human genome

**Fig. 5:**
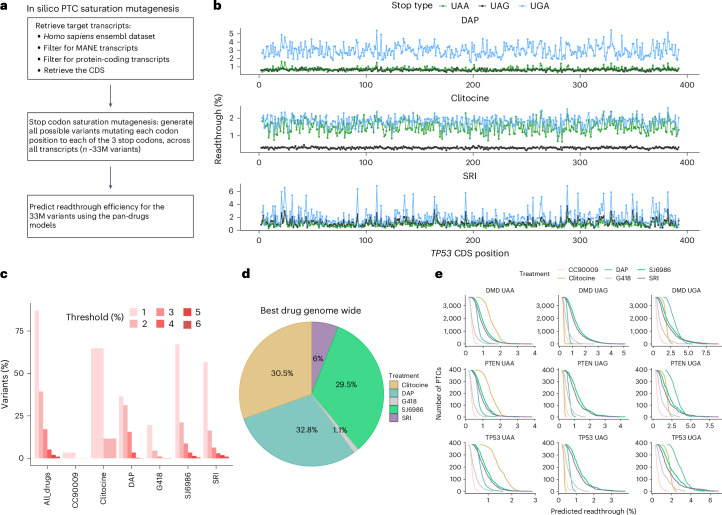
In silico nonsense saturation mutagenesis of the human genome. **a**, Generation of the comprehensive in silico dataset with all possible nonsense mutations in human coding genes. **b**, Readthrough predictions along the coding sequence (CDS) of *TP53* for each stop codon type. Each panel represents a drug-specific readthrough prediction—DAP (top), clitocine (middle) and SRI (bottom). **c**, Percentage of variants genome-wide with readthrough over a given threshold (color legend) for each drug separately and when considering all eight drugs together (All_drugs). **d**, Percentage of the number of variants across all possible variants in the human exome for which each drug is predicted to display the highest readthrough efficiency. **e**, Cumulative histograms showing the number of variants as a function of readthrough efficiency for the genes *DMD* (top), *PTEN* (middle) and *TP53* (bottom), stratified by stop codon type as UAA (left), UAG (center) and UGA (right). Source data

**Extended Data Fig. 5:**
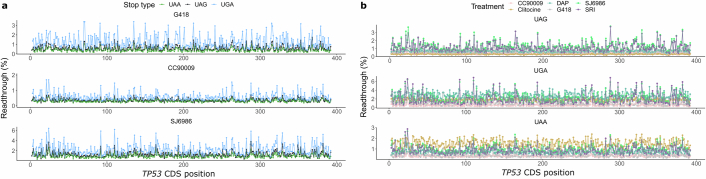
In silico PTC saturation mutagenesis. **a**, Overview of the readthrough predictions along the coding sequence of *TP53* for each stop codon type. Each panel represents a drug-specific prediction: G418 (top), CC90009 (middle) and SJ6986 (bottom). **b**, Same as **a**, but drugs are represented as colors and each panel belongs to a different stop type. Source data

The accurate prediction of drug-induced readthrough by our interpretable model allows us to provide readthrough predictions for every possible PTC in every transcript of the human genome (Fig. 5a). In total, one to three nucleotide substitutions can introduce 32.7 million stop codons in the 19,061 human protein-coding transcripts (Ensembl v107 genes, hg38 assembly), and we made readthrough predictions for six drugs, available as a resource named RTDetective that can be visualized along the human genome using the UCSC browser (Fig. 5b and Extended Data Fig. 5a,b; 10.6084/m9.figshare.23708901).

We estimate that by using these six drugs, a readthrough of >2% can be achieved for 13 million of 32.7 million (39.6%) possible stops in the human genome, with a readthrough of >1% possible for 28.6 million stops (87.3%; Fig. 5c). The individual drugs are predicted to result in >2% readthrough for 31.4%, 21.3%, 16.2%, 11.7%, 4.3% and 0.02% of PTCs for DAP, SJ6986, SRI, clitocine, G418 and CC90009, respectively. Clitocine is a mid-intensity readthrough drug but spans UAA and UGA stops, inducing 1.5–2% readthrough for many variants but higher readthrough for only a few. Considering all 32.7 million possible PTCs, the most effective drug in 32.8% of cases is DAP, followed by clitocine (30.5%), SJ6986 (29.5%), SRI (6%) and G418 (1.1%; Fig. 5d).

Some drug-stop codon type combinations show promising potential, as illustrated for *DMD*, *PTEN* and *TP53* (Fig. 5e). DAP stimulates readthrough >2% for almost 100% of the UGA variants across the three genes. For UAA mutations, clitocine emerges as the best candidate (readthrough >1.5% for ~50% of PTCs). For UAG mutants, the recently reported eRF1/eRF3 inhibitors SRI-41315 and SJ6986 (refs. ^25,30^) show promise (across *DMD*, *PTEN* and *TP53*, readthrough > 1.5% for 33% and 25% of UAG PTCs for SJ6986 and SRI, respectively, whereas readthrough > 1.5% for <0.1% of UAG PTCs for clitocine and DAP). Thus, even for UAG variants that have been considered particularly difficult to suppress, drug-induced readthrough provides a promising therapeutic strategy, provided that the correct drugs are matched to each PTC.

### Readthrough of natural termination codons

Drug-induced readthrough over natural termination codons (NTCs) has been postulated as the main cause of toxicity observed in patients^49^. Notably, translation termination of NTCs differs from that of PTCs, with additional elements involved, including the proximity to Poly(A) tails^50^, readthrough peptide targeting pathways^51^ and in-frame downstream 3′-UTR stops that together reduce the generation of readthrough peptides at NTCs. Here we focus on the contribution of local sequence context to NTC readthrough, while the additional elements listed above remain to be addressed in future work.

**Fig. 6:**
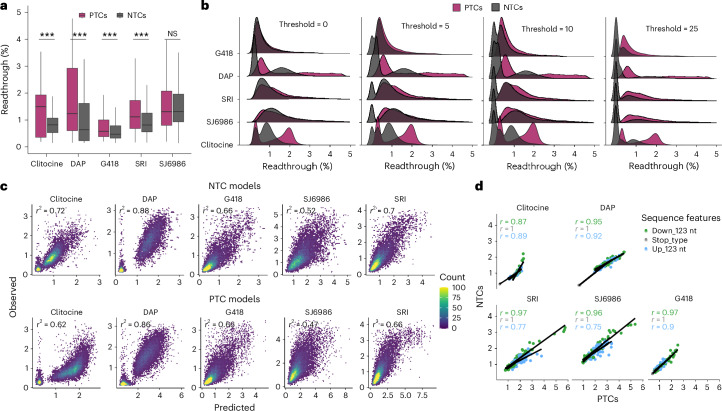
Quantifying readthrough for >17,000 natural termination codons (NTCs). **a**, Readthrough distributions across drugs for the PTC and NTC libraries (two-sided Wilcoxon test, ***P < 2 × 10^−16^ and *n* = 23,459, *n* = 23,096, *n* = 22,905, *n* = 22,989 for clitocine, DAP, G418 and SRI, respectively, whereas *P* = NS and *n* = 23,201 for SJ6986). The top and bottom sides of the box are the lower and upper quartiles. The box covers the interquartile interval, where 50% of the data are found. The horizontal line that splits the box in two is the median. **b**, Readthrough distributions across drugs for the PTC and NTC libraries. The threshold indicates the number of amino acids downstream of the NTC considered for the analysis. NTC variants with a 3′-UTR in-frame stop codon more proximal than the threshold are assumed to have a readthrough of 0%. Increasing the threshold increases the number of readthrough-insensitive variants. The number of NTC high-confidence variants (≥10 reads) recovered for each treatment and for which readthrough percentages were quantified are 17,812, 17,654, 17,382, 17,661 and 17,587 for clitocine, DAP, G418, SJ6986 and SRI, respectively. **c**, Drug-specific models predictive performance on the NTC dataset using NTC-trained tenfold cross-validated models (top) or PTC-trained models (bottom). **d**, Correlation of the mean readthrough for each sequence context between PTCs and NTCs, colored by the sequence feature. NS, not significant. Source data

We leveraged our system to assess the readthrough stimulation of five high-readthrough drugs (clitocine, DAP, G418, SJ6986 and SRI) over NTCs from ~18.8k human protein-coding genes, preserving the upstream and downstream (3′ UTR) 66 nts, but selectively removing the in-frame stops downstream (to uniquely assess the role of the stop codon proximal sequence to NTC readthrough). Readthrough measurements were highly correlated across replicates (*r* = 0.88–0.99), and on average, 5 million cells were sorted (~277 cells per variant) and 17,590 high-confidence variants (≥10 reads) were recovered in each experiment (Supplementary Table 11). Distributions resembled their PTC counterparts with somewhat lower readthrough (except for SJ8986), with the largest PTC–NTC differences for DAP (1.9-fold, adjusted *P* < 2 × 10^−16^) and SRI (1.5-fold, adjusted *P* < 2 × 10^−16^) drugs (Fig. 6a). This suggests a modest readthrough-protective effect of the NTC-surrounding sequence, even after removing additional downstream stops. Artificially setting the readthrough of variants whose endogenous gene has downstream in-frame stops to 0% (assuming that readthrough over two stops is ~0%), shows a strong drop in readthrough of the NTC population (Fig. 6b).

Finally, we used the drug-induced readthrough of the NTC library as an additional test of our PTC-trained predictive models (Fig. 6c). Performance was good across drugs (*r* = 0.47–0.86) and similar to that for new models trained on the NTC data itself (*r* = 0.52–0.88 by tenfold cross-validation; Supplementary Table 12). Indeed, the mean readthrough for each sequence feature correlates very well in PTCs versus NTCs, supporting a similar sequence context role in both translation termination scenarios (Fig. 6d).

## Discussion

We have presented here a systematic quantification of drug-induced stop codon suppression comprising >140,000 readthrough measurements in human cells made using eight drugs. Our massively parallel assay represents a substantial scaling-up of data production compared to previous studies^14,16^, generating datasets of sufficient size to train models to accurately predict drug-induced nonsense suppression genome-wide.

Our results show that each drug only induces the readthrough of a subset of pathogenic PTCs, and our models use the sequence context to predict the readthrough specificity of each drug. This diversity of drug responses means that for any particular disease gene, there is no single drug that triggers a strong readthrough of all pathogenic PTCs. Rather, effective clinical nonsense suppression will require a panel of drugs, with the appropriate drug selected for each patient according to the identity of the PTC that they carry.

The models that we have trained are deliberately interpretable and relatively simple, and yet they explain 94% of the variance in our dataset (excluding FUr and gentamicin). It is possible that black box machine learning models may further improve predictive performance, but model interpretability both aids mechanistic understanding and is desirable for models to be used in clinical decision-making^52^.

We envisage that accurate genome-scale prediction of drug-induced readthrough will improve clinical trial design and the development of personalized nonsense suppression therapies. To date, trial designs have nearly all ignored the large variation in readthrough efficacy across PTCs^45^, resulting in suboptimal matching between patient PTCs and drugs. The use of the right drugs but in the wrong patients is, in retrospect, likely to have been an important cause of trial failure.

Our general approach can be used to rapidly quantify the specificities of new nonsense suppression therapeutics, allowing their clinical efficacy to then be tested in the subset of patients in which they are likely to be most effective.

Our study has some important limitations. First, readthrough was not measured in the endogenous genomic context, and long-distance features may also affect readthrough. Second, not all readthrough translation products will be functional proteins. Third, the level of readthrough required for clinical benefit will vary across proteins and diseases. Finally, the clinical efficacy of readthrough-promoting drugs will depend on additional parameters such as pharmacokinetics, pharmacodynamics and drug toxicity.

Taken together, our results show that the specificities of nonsense suppression therapies differ extensively across drugs, and these specificities can be rapidly learned using high-throughput experiments to allow accurate prediction of drug responses. Looking forward, we believe that the goal should be to develop an expanding portfolio of readthrough drugs with defined and complementary specificities such that effective and specific nonsense suppression therapy can be achieved for any pathogenic stop codon in the human genome.

## Methods

### Ethics and consent

The study did not require any specific ethics approval. It builds on publicly available data provided by ClinVar, TCGA and MSK-IMPACT. The patient data obtained from two clinical trials were already released.

### Library design

For the PTCs library, genetic disease and cancer germline variants were retrieved (*n* = 3498) from the ClinVar database, where all pathogenic nonsense variants whose review status was two or more stars were included (a final filtering step was carried out to decrease the penetrance of overrepresented genes such as BRCA2). Somatic cancer variants were obtained from MSK-IMPACT and TCGA databases with at least two entries in either dataset (*n* = 2,372). Finally, a control no-nonsense *TP53* variant (c.541C-c.687T nucleotides from ENST00000269305 transcript) was included to use as a 100% readthrough control to normalize the expression of the PTC variants and get estimates for the percentage of WT protein expression for each variant. The 147 nt spliced-mRNA context of each nonsense variant (MANE transcript isoform) was retrieved from Ensembl (v104) to preserve a large sequence context (72 nts upstream and 72 nts downstream).

For the NTCs library, the 66 nts upstream and downstream (3′ UTR) for the spliced MANE isoform of all 18,824 human protein-coding genes were retrieved from Ensembl (v104). We artificially performed 1-Hamming distance substitutions to remove all in-frame 3′-UTR stop codons (TAA->TAC, TAG, TGA->TGG) to assess the role of sequence context on readthrough without the confounding influence of tandem stops. The no-nonsense *TP53* variant (c.541C-c.687T nucleotides from ENST00000269305 transcript) was included to use as a 100% readthrough control.

No statistical methods were used to predetermine sample sizes of the libraries, but our sample sizes are 100–1,000 folds larger than those reported in previous publications^11–14,16^.

### Readthrough reporter

We designed a double fluorescent reporter plasmid (pIT092) to quantify readthrough. The plasmid encodes a single transcript that contains the open reading frames (ORFs) of EGFP, T2A (2×) and mCherry from 5′–3′, respectively. The library oligo pool was cloned in-frame between the two T2A sequences. T2As allow the independent folding of the fluorescent proteins and prevent undesired effects of the variable sequence on their folding and stability. In a normal translation event, termination occurs in the PTC of the library, protecting mCherry from translation. However, if readthrough occurs, the ribosome extends elongation until the mCherry stop codon translating the mCherry protein along the way. Hence, mCherry fluorescence is proportional to readthrough efficiency, and we used it as our assay readout. EGFP is used to filter out those cells that either do not have EGFP or have unexpectedly high levels of EGFP. These cells are likely to have either aberrant cloning, out-of-frame integration, promoter mutations, promoter silencing, transcript-stability mutations, etc., and might be misleading if included in the assay. For some of the treatments, we detect a slight EGFP increase in the mCherry^+^ population, suggesting a readthrough-mediated transcript stabilization either via NMD inhibition or translation-mediated mRNA protection. In those cases, the mCherry increase (in mCherry^+^ versus mCherry^−^ populations) is higher than the EGFP increase, proving that the drugs are increasing the mCherry signal via readthrough. pIT092 is suitable for genomic integration using the HEK293T landing pad (LP) system^19^, which ensures that each cell integrates only one variant providing a direct genotype–phenotype linkage. The vector contains BxBI-compatible attB sites that allow recombination into the genomic LP of HEK293T_LP cells. After genomic integration into the LP locus, the ORF sequence is placed right downstream of a tetracycline induction cassette, allowing its expression when doxycycline is added to the media.

### Library cloning

Oligos were ordered as an oligo pool to Twist Biosciences containing the variable part (library) and two constant sequencesfor PCR amplification and subcloning. The oligo pool was PCR-amplified for 14 cycles using primers oIT204 and oIT340 (Supplementary Table 14). The oligo pool was cloned between the EGFP-T2A and T2A-mCherry ORFs of pIT092 using Gibson Assembly. The library was electrotransformed using Neb10 electrocompetent bacteria and grown in 100 ml overnight culture. Library complexity and representativity of the variants were estimated by plating a small amount of the transformation reaction and extrapolating the total number of transformants. Individual clones were Sanger sequenced to confirm the expected structure and diversity.

### Stable cell line generation

To generate the cell lines, we used the HEK293T_LP cell line generated in ref. ^19^ (TetBxB1BFP-iCasp-Blast Clone 12 HEK293T cells), which allows the stable single-copy integration of variants in the genome. Mutational libraries cloned into the LP compatible construct (pIT092) are cotransfected (1:1) with a BxBI expression construct (pCAG-NLS-Bxb1) into the HEK293T_LP cell line using lipofectamine 3000 according to the manufacturer’s instructions in three T150 cm^2^ flasks. This cell line has a genetically integrated tetracycline induction cassette, followed by a BxBI recombination site and a split rapalog-inducible dimerizable Casp-9. Cells were maintained in DMEM supplemented with 10% FBS tetracycline-free without antibiotics. Two days after transfection, doxycycline (2 μg ml^−1^; Sigma-Aldrich) was added to induce expression of the library (recombined cells) or the iCasp-9 protein (no recombination). Twenty-four hours later, 10 nM rimiducid (Selleckchem, AP1903) was added to the cells. Successful recombination frameshifts the iCasp-9 out of frame. However, nonrecombined cells express iCasp-9, which dimerizes in the presence of rimiducid and induces apoptosis. One day after rimiducid treatment, the media was changed back to DMEM + doxycycline, and cells were maintained in culture for the following 5 days to obtain a large volume of cells for downstream experiments and cryostorage.

### Readthrough compounds

We tested a panel of 20 compounds reported to have readthrough activity (Supplementary Table 2), in our library-integrated HEK293T_LP cells. If a drug induces readthrough, the fluorescence-activated cell sorting (FACS) profile would be different than the untreated cells; specifically, we would observe an increase in the mCherry^+^ population (Extended Data Fig. 1a). Readthrough was calculated as the (mCherry^+^ and EGFP^+^)/(EGFP^+^) cell ratio multiplied by the mean mCherry intensity of the mCherry^+^ population and normalized to the readthrough of the no-nonsense variant (Extended Data Fig. 1d). All drugs were tested at four or more different concentrations ranging along orders of magnitude, to ensure that a negative result was not due to a concentration-related problem. The 4 × 10^5^ library-integrated HEK293T_LP cells were seeded in six-well plates and treated with 2 μg ml^−1^ doxy to induce the expression of the transcript, and after 24 h, the drug was added to the medium. Readthrough was measured 48 h after treatment with the BD LSRFortessa Cell Analyzer as described above. Eight drugs, namely SRI, clitocine, SJ6986, DAP, G418, gentamicin, CC90009 and FUr, were validated, whereas the remaining 12 did not trigger detectable readthrough in our system at the tested concentrations. We did toxicity titrations for the eight positive drugs (Extended Data Fig. 1c). In total, 2 × 10^4^ cells were plated in 96-well plates, and the CellTiter-Glo Luminescent Cell Viability Assay (Promega) was used to quantify cell viability 48 h after drug or vehicle treatment using a Tecan Infinite M Plex plate reader (Tecan). For each drug, the concentration that didn’t decrease cell viability by more than 25% and exhibited the highest readthrough was selected (Extended Data Fig. 1c,d). For G418, we chose a concentration that dropped cell viability by a slightly different quantity of 30%, because the increase in readthrough was substantial, compared to the concentration that reduced viability by 25%. Also, note that very high concentrations of gentamicin and G418 were used to counteract the effect of the aminoglycoside-resistance cassette of the HEK293T_LP cells^19^.

The validated drugs comprise different classes of small molecules. G418 and gentamicin bind to the decoding center of the small ribosomal subunit; SRI, SJ6986 and CC90009 are eRF1/eRF3 inhibitors; DAP interferes with the activity of a tRNA-specific 2′-O-methyltransferase (FTSJ1); and clitocine and FUr are nucleotide analogs that get incorporated into the mRNA. Some were reported decades ago, and their readthrough potential is supported by extensive literature (gentamicin, G418) and tested in several clinical trials (most of them with disappointing and confusing outcomes). In contrast, others have been recently described as drugs, and little is known about their readthrough stimulatory potential.

### FACS

Cells were grown on standard culture plates in tetracycline-free DMEM supplemented with 10% FBS, and without antibiotics. They were split before reaching confluency to maintain cell health. Cells were detached with trypsin, spun down and washed with PBS. For the sort-seq experiments^53^, cells were treated with 2 μg ml^−1^ doxy to induce the expression of the transcript, and after 24 h, the drug was added to the medium for 48 h more. We used high volumes of cells to ensure that each variant was represented >100 times in the cell population.

Cells were sorted on a BD Influx Cell Sorter and analyzed with BD FACS Software (1.0.0.650). Cells were gated by forward scattering area and by side scattering area to retain whole cells, forward scattering width and height to discard aggregates, and by DAPI staining to retain only recombined and alive cells. EGFP and mCherry fluorescence were excited with 488 nm and 561 nm lasers and recorded with 530/40 bandpass (BP) and 593/40 BP channels, respectively. EGFP^+^ cells were sorted based on mCherry expression into three to five populations (Extended Data Fig. 1a), depending on how stretched the library was across the mCherry axis. The top-right population in the Extended Data Fig. 1a plots represents the no-nonsense variant and was sequenced for one drug condition (gentamicin) of the natural stops library. In total, 30% of the reads in that population indeed map to the no-nonsense *TP53* variant, in contrast to <0.01% in the other populations (Extended Data Fig. 1g). For most of the populations, 400k cells were sorted. However, for some minor populations representing <2% of the total population, we sorted less cells (100–200k). The percentage of cells in each bin population was used for normalization during sequencing analysis (see below). Experiments were performed in biological duplicates, and, on average, 1.6 million cells were sorted in each drug experiment (~272 cells per variant).

### DNA extraction

Sorted cells were centrifuged at 1,200 rpm for 3 min, and the pellet was used to conduct genomic DNA extraction following the DNeasy Blood & Tissue Kit (Qiagen) and resuspended in 80 μl of Milli-Q water.

### Sequencing library preparation

The sequencing libraries were constructed in three consecutive PCR reactions. The first PCR intends to amplify the library fragment from the genomic DNA pool without amplifying the remaining plasmid from transfection. It uses a forward (oIT314) primer annealing in the LP outside of the recombined sequence and a reverse (oIT205) primer annealing at the 3′ end of the library fragment. This ensures that plasmid DNA is not amplified because it lacks the annealing site for the forward primer. The second PCR (PCR2) was designed to insert part of the Illumina adapters and to increase the nucleotide complexity of the first sequenced bases by introducing frame-shift bases between the adapters and the sequencing region of interest. The third PCR (PCR3) was necessary to add the remainder of the Illumina adapter and the demultiplexing indexes. All PCRs were run using Q5 Hot Start High-Fidelity DNA Polymerase (New England Biolabs) according to the manufacturer’s protocol.

All genomic DNA extracted from each bin was used as a template for PCR1 and amplified using 25 pmol of primers oIT205 and oIT314. The annealing temperature was set to 66 °C, extension time to 1 min and number of cycles to 25. Because high volumes of genomic DNA inhibit PCR reactions, we aliquoted each sample in eight PCRs and ran them in 96-well plates. Excess primers were removed by adding 0.04 μl of ExoSAP-IT (Affymetrix) per microliter of PCR1 reaction and incubated for 20 min at 37 °C followed by an inactivation for 15 min at 80 °C. Then, the PCRs of each sample were pooled together and purified using the MinElute PCR Purification Kit (Qiagen) according to the manufacturer’s protocol. DNA was eluted in Milli-Q water to a volume of 20 μl.

In total, 2 μl of PCR1 product were used as template for PCR2, together with 25 pmol of pooled frame-shift primers (oIT_ILL_204_mix and oIT_ILL_205_mix; Supplementary Table 14). The PCR reactions were set to an annealing temperature of 66 ºC, 15 s of extension time and were run for eight cycles. Excess primers were removed by adding 0.04 μl of ExoSAP-IT (Affymetrix) per microliter of PCR1 reaction and incubated for 20 min at 37 °C followed by an inactivation for 15 min at 80 °C. The PCRs of each sample were purified using the MinElute PCR Purification Kit (Qiagen) according to the manufacturer’s protocol. DNA was eluted in Milli-Q water to a volume of 10 μl.

In total, 2 μl of PCR2 products were used as template for PCR3. In PCR3, the remaining parts of the Illumina adapters were added to the library amplicon. The forward primer (oIT_GJJ_1J) was the same for all samples, while the reverse primer (oIT_GJJ_2J) differed by the barcode index, to allow pooling of all samples together and demultiplexing after deep sequencing (Supplementary Table 14). Eight cycles of PCR3s were run at 62 °C of annealing temperature and 25 s of extension time. All reactions from the same sample were pooled together, and an aliquot was run on a 2% agarose gel to be quantified. After quantification, samples with different Illumina indexes that were sequenced together in the same flow cell were pooled in an equimolar ratio, run on a gel and purified using the QIAEX II Gel Extraction Kit. The purified amplicon library pools were subjected to 150-bp paired-end NextSeq 500 sequencing (Illumina) at the CRG Genomics Core Facility.

### Sequencing data processing

FastQ files from paired-end sequencing of all experiments were processed with DiMSum (v.1.3; https://github.com/lehner-lab/DiMSum) to obtain the read counts for each variant. DimSum applies stringent quality filters to discard low-quality reads, reads with sequencing errors, etc., to ensure that only high-quality reads are used for downstream analysis.

The DimSum output read count tables were used to calculate readthrough estimates for each variant as follows: the read count table provides the distribution of each variant among the different sorting gates. Because gates harbored different percentages of the general population, they had to be sorted at different times to get the same number of cells in each bin, forcing us to calculate the mCherry distributions in relative numbers (formula 1 in Supplementary Note 8). The distribution of each variant is generated by calculating its proportion of reads in each sorting gate (*j*), with *m* being the total number of sorting gates. All reads of a given variant in a given sorting gate (*r*_*j*_) are (1) divided by the total number of reads of that gate (*R*_*j*_) yielding a normalized reads value, (2) multiplied by a fixed value (*pc*) corresponding to the percentage of cells of the total population that belong to that gate and (3) and multiplied by a second fixed value (*fv*) corresponding to the mean mCherry signal of the gate. Finally, (4) it is averaged by the total number of normalized reads across gates (*N*). In steps (1) and (2), we are simply calculating the percentage of reads of the total population belonging to each variant in each gate ($\frac{{r}_{j}}{{R}_{j}}{{\mathrm{pc}}}_{j}$). In steps (3) and (4) we obtain a value corresponding to a normalized mean mCherry value for each variant. Then, by dividing this mCherry value of each variant by the mCherry value of the untreated no-nonsense variant (which represents the amount of WT protein in basal conditions), the readthrough percentage for each variant (RTp, percentage of WT protein expression) is calculated (formula 2 in Supplementary Note 8). The no-nonsense population does not undergo readthrough and displays the same mCherry value across treatments, as expected. However, under clitocine conditions, its mCherry signal is 1.6-fold higher, suggesting an RNA stabilization effect of clitocine. The normalization with the no-nonsense variant in the untreated condition sets all the treatments’ readthrough distributions on the same scale and allows direct comparison across drugs. The EGFP^−^/mCherry^−^ population was used to calibrate the voltage of the sorting instrument across treatments and replicates. The standard deviation (s.d.) from the two replicates was used as the error measure. Readthrough percentage efficiencies for all variants and drugs are included in Supplementary Tables 3 (PTCs) and 11 (NTCs). Experiments were performed in biological duplicates, and, on average, 5,602 high-confidence variants (≥10 reads) were recovered in each experiment. We retained variants with ≥10 reads. Shapiro–Wilk test was used to determine the non-normality of the sample, and accordingly, nonparametric tests (Kruskal–Wallis and Wilcoxon signed-rank tests) were used to assess significance. Note that the two clitocine replicates were sigmoidally related due to a difference in the voltage setting of the flow cytometer between experiments. A LOESS model was fit and used to predict replicate 2 based on replicate 1. This transformation yielded a linear relation between the two replicates.

### Single-variant validation experiments

To validate the assay, we set out to individually measure the readthrough of 15 variants. We selected 15 variants spanning the whole dynamic range when treated with SRI plus the no-nonsense control and individually cloned and integrated them into HEK293T_LP, yielding 16 stable cell lines each expressing a different variant. In parallel, we conducted the same measurements in MCF7 and HeLa cell lines (cells were maintained in tetracycline-free DMEM supplemented with 10% FBS without antibiotics). Because the LP is unique to HEK293T cells, we used transient transfection to express the readthrough variants in MCF7 and HeLa cell lines. Constructs harboring the 16 variants were cloned out from pIT092 and cloned in a mammalian CMV-expression vector (pIT075).

HEK293T_LP were treated with 2 μg ml^−1^ doxy, and 50 ng of pIT075 plasmids were transfected into MCF7 and HeLa with lipofectamine 3000. Twenty-four hours later, cells were treated with SRI at the same concentration than used in the DMS assay (7.5 μM). 48 h after treatment, EGFP and mCherry fluorescence were quantified using 530/40 BP and 593/40 BP channels in the BD LSRFortessa Cell Analyzer. EGFP^+^ cells were used to calculate the readthrough by multiplying the percentage of mCherry^+^ cells by its mean mCherry intensity and finally normalizing to the no-nonsense variant (formula 3 in Supplementary Note 8). We termed these readthrough values as ‘readthrough *P*_individual_’ because they refer to individual measurements of readthrough percentages of each variant. Readthrough *P*_individual_ were correlated against the readthrough *P*_DMS_ estimates of the 15 variants to calculate the correlation coefficient between our DMS assay and individually measured readthrough (Supplementary Table 1).

### Sequence features

We listed a large set of sequence features to test their contribution to readthrough variability. Features were chosen based on literature and preliminary results, but we also listed several features for which we had no evidence of their participation in readthrough. All features tested in the predictive models can be found in Supplementary Table 4. See Supplementary Note 6 for extended information on model design.

### tAI

The tAI is a measure of translational efficiency that takes into account the intracellular concentration of tRNA molecules and the efficiencies of each codon–anticodon pairing^41,42^. The pairing affinity of each codon–anticodon is specific to each species. The human-specific tAI indexes were downloaded from the STADIUM database as of January 2023 (ref. ^54^). tAI for a given sequence was calculated as the mean tAI across all codons of the sequence.

### CAI

The CAI is an estimate of translational efficiency based on the similarity of codon usage of one sequence with regard to the genome codon usage^40^. The human codon usage table was downloaded from the Codon/Codon Pair Usage Tables project release as of January 2023 (ref. ^55^). CAI for a given sequence was calculated as the mean CAI across all codons of the sequence.

### In silico saturation mutagenesis

We used the general drug model to perform an in silico prediction of the readthrough efficiency of all possible nonsense mutations in the human exome, resulting in 32.7 × 10^6^ predictions for the 19,061 protein-coding transcripts (Ensembl v107 genes, hg38 assembly) for each drug. For each codon position of each protein-coding transcript, a readthrough efficiency value was estimated for each drug.

### Statistics and reproducibility

Statistical tests were performed in R (v4.3.1) using RStudio (v2023.03.0+386). Kruskal–Wallis tests were used to assess the general association of sequence features and readthrough, whereas Wilcoxon tests were used for pairwise comparisons of specific levels of the sequence features. Wilcoxon tests were either one- or two-sided, and this information is always provided in the figure legend. *P* values were adjusted using the p.adjust function in R (Benjamini–Hochberg). No statistical method was used to predetermine the sample size. Variants with <10 reads were not used for analyses, and analyses included all other variants unless explicitly indicated. No blinding or randomization was performed.

### Reporting summary

Further information on research design is available in the Nature Portfolio Reporting Summary linked to this article.

## Online content

Any methods, additional references, Nature Portfolio reporting summaries, source data, extended data, supplementary information, acknowledgements, peer review information; details of author contributions and competing interests; and statements of data and code availability are available at 10.1038/s41588-024-01878-5.

## Supplementary information

Supplementary Information Supplementary Notes 1–8 and Supplementary Fig. 1.

Reporting Summary

Supplementary Data Supporting data for Supplementary Fig. 1.

Supplementary Tables Supplementary Table 1: Single-variant validation experiments. DMS and individual SRI-stimulated readthrough measurements for the 25 variants used for validation experiments. The s.d. across two replicates is provided. The ‘upper_limit_variant’ column indicates if the variant was tested on the first validation round or on the second round (to specifically define the upper limit of the assay). Supplementary Table 2: Readthrough compounds. All readthrough compounds were tested, along with the concentration and solvent used and the company/lab they were obtained from. For the compounds whose reported readthrough stimulation could not be reproduced in our assay (validated = ‘no’), the range of concentrations tested is provided. Supplementary Table 3: PTC dataset. Readthrough measurements for all PTC variants across all drugs and the untreated condition. Supplementary Table 4: Sequence features tested for readthrough association. All 47 sequence features whose role in readthrough regulation was tested during model generation. The first column shows the feature short name used throughout the paper, whereas the second column provides a more detailed explanation. Supplementary Table 5: TP53 and PTEN mutants. Readthrough measurements across all conditions for the nine highly prevalent PTEN (*n* = 4) and TP53 (*n* = 5) mutants addressed in Fig. 3d and Extended Data Fig. 3f. Supplementary Table 6: Clinical trials. A list of the 42 clinical trials testing readthrough compounds is revised in this manuscript. Relevant information such as the drug tested, the patient’s pathology, the number, age and gender of patients, the clinical phase and the availability of mutational data are provided. Supplementary Table 7: Predictions for the PTC drug-specific models. Observed and predicted readthrough measurements obtained across the ten rounds of cross-validation (90–10% training-testing data) during model training. The ‘treatment’ column indicates the PTC drug-specific model. Supplementary Table 8: Predictions for the PTC pan-drug model. Observed and predicted readthrough measurements obtained across the ten rounds of cross-validation (90–10% training-testing data) during model training for the PTC pan-drug model. Supplementary Table 9: PTC drug-specific model coefficients. Model coefficients for the PTC drug-specific models. The ‘fold’ columns show the coefficients obtained in each cross-validation round. The ‘mean’, ‘s.d.’ and ‘ci95’ columns show the mean, s.d. and 95% confidence intervals across the tenfolds. A two-sided *t* test was performed to interrogate the null hypothesis that the coefficient equals 0. *P* values and adjusted *P* values (false discovery rate (FDR) = 0.05) are shown. The ‘feature’ and ‘terms’ columns indicate the sequence feature and specific feature level, respectively. Supplementary Table 10: PTC pan-drug model coefficients. Model coefficients for the PTC pan-drug model. The ‘fold’ columns show the coefficients obtained in each cross-validation round. The ‘mean’, ‘s.d.’ and ‘ci95’ columns show the mean, s.d. and 95% confidence intervals across the tenfolds. A two-sided *t* test was performed to interrogate the null hypothesis that the coefficient equals 0. *P* values and adjusted *P* values (FDR = 0.05) are shown. The ‘feature’ and ‘terms’ columns indicate the sequence feature and specific feature level, respectively. Supplementary Table 11: NTC dataset. Readthrough measurements for all NTC variants across all drugs and the untreated condition. Supplementary Table 12: Predictions for the NTC drug-specific models. Observed and predicted readthrough measurements obtained across the ten rounds of cross-validation (90–10% training-testing data) during model training. The ‘treatment’ column indicates the NTC drug-specific model. Supplementary Table 13: NTC drug-specific model coefficients. Model coefficients for the NTC drug-specific models. The ‘fold’ columns show the coefficients obtained in each cross-validation round. The ‘mean’, ‘s.d.’ and ‘ci95’ columns show the mean, s.d. and 95% confidence intervals across the tenfolds. A two-sided *t* test was performed to interrogate the null hypothesis that the coefficient equals 0. *P* values and adjusted *P* values (FDR = 0.05) are shown. The ‘feature’ and ‘terms’ columns indicate the sequence feature and specific feature level, respectively. Supplementary Table 14: Primers. List of primers used for plasmid generation and library preparation.

## Source data

Source Data Fig. 1 Statistical source data.

Source Data Fig. 2 Statistical source data.

Source Data Fig. 3 Statistical source data.

Source Data Fig. 4 Statistical source data.

Source Data Fig. 5 Statistical source data.

Source Data Fig. 6 Statistical source data.

Source Data Extended Data Fig. 1 Statistical source data.

Source Data Extended Data Fig. 2 Statistical source data.

Source Data Extended Data Fig. 3 Statistical source data.

Source Data Extended Data Fig. 4 Statistical source data.

Source Data Extended Data Fig. 5 Statistical source data.

## Data Availability

All DNA sequencing data have been deposited in the Sequence Read Archive with accessions PRJNA996618 (PTCs) and PRJNA1073909 (NTCs). The readthrough efficiency predictions have been made available through the Figshare repository at https://figshare.com/articles/dataset/Readthrough_predictions/23708901 and via a digital object identifier (10.6084/m9.figshare.23708901). All readthrough measurements are provided in Supplementary Tables 3 and 11. The MSK-IMPACT and TCGA datasets were downloaded from cBioPortal (https://www.cbioportal.org/) on 2 June 2021. The ClinVar dataset was downloaded from https://ftp.ncbi.nlm.nih.gov/pub/clinvar/vcf_GRCh38/ on 3 June 2021. Source data are provided with this paper.

## Code availability

Source code used to perform all analyses and to reproduce all figures in this work is available at GitHub (https://github.com/lehner-lab/Stop_codon_readthrough) and has been archived to Zenodo (https://zenodo.org/records/12698349)^56^.

## References

[1] Landrum, M. J. et al. ClinVar: improving access to variant interpretations and supporting evidence. *Nucleic Acids Res.* **46**, D1062–D1067 (2018). 29165669 10.1093/nar/gkx1153 PMC5753237

[2] Stenson, P. D. et al. The Human Gene Mutation Database (HGMD®): optimizing its use in a clinical diagnostic or research setting. *Hum. Genet.* **139**, 1197–1207 (2020). 32596782 10.1007/s00439-020-02199-3 PMC7497289

[3] Supek, F., Lehner, B. & Lindeboom, R. G. H. To NMD or not to NMD: nonsense-mediated mRNA decay in cancer and other genetic diseases. *Trends Genet.* **37**, 657–668 (2021). 33277042 10.1016/j.tig.2020.11.002

[4] Lykke-Andersen, S. & Jensen, T. H. Nonsense-mediated mRNA decay: an intricate machinery that shapes transcriptomes. *Nat. Rev. Mol. Cell Biol.* **16**, 665–677 (2015). 26397022 10.1038/nrm4063

[5] Lombardi, S., Testa, M. F., Pinotti, M. & Branchini, A. Molecular insights into determinants of translational readthrough and implications for nonsense suppression approaches. *Int. J. Mol. Sci.* **21**, 9449 (2020). 33322589 10.3390/ijms21249449 PMC7764779

[6] Dabrowski, M., Bukowy-Bieryllo, Z. & Zietkiewicz, E. Advances in therapeutic use of a drug-stimulated translational readthrough of premature termination codons. *Mol. Med.* **24**, 25 (2018). 30134808 10.1186/s10020-018-0024-7 PMC6016875

[7] Gunn, G. et al. Long-term nonsense suppression therapy moderates MPS I-H disease progression. *Mol. Genet. Metab.* **111**, 374–381 (2014). 24411223 10.1016/j.ymgme.2013.12.007 PMC3943726

[8] Palomar-Siles, M. et al. Translational readthrough of nonsense mutant TP53 by mRNA incorporation of 5-fluorouridine. *Cell Death Dis.* **13**, 997 (2022). 36433934 10.1038/s41419-022-05431-2 PMC9700717

[9] Sarkar, C., Zhang, Z. & Mukherjee, A. B. Stop codon read-through with PTC124 induces palmitoyl-protein thioesterase-1 activity, reduces thioester load and suppresses apoptosis in cultured cells from INCL patients. *Mol. Genet. Metab.* **104**, 338–345 (2011). 21704547 10.1016/j.ymgme.2011.05.021 PMC3220191

[10] Ramalho, A. S. et al. Five percent of normal cystic fibrosis transmembrane conductance regulator mRNA ameliorates the severity of pulmonary disease in cystic fibrosis. *Am. J. Respir. Cell Mol. Biol.* **27**, 619–627 (2002). 12397022 10.1165/rcmb.2001-0004OC

[11] Welch, E. M. et al. PTC124 targets genetic disorders caused by nonsense mutations. *Nature* **447**, 87–91 (2007). 17450125 10.1038/nature05756

[12] Trzaska, C. et al. 2,6-Diaminopurine as a highly potent corrector of UGA nonsense mutations. *Nat. Commun.* **11**, 1509 (2020). 32198346 10.1038/s41467-020-15140-z PMC7083880

[13] Floquet, C., Hatin, I., Rousset, J.-P. & Bidou, L. Statistical analysis of readthrough levels for nonsense mutations in mammalian cells reveals a major determinant of response to gentamicin. *PLoS Genet.* **8**, e1002608 (2012). 22479203 10.1371/journal.pgen.1002608 PMC3315467

[14] Pranke, I. M. et al. The U UGA C sequence provides a favorable context to ELX-02 induced CFTR readthrough. *J. Cyst. Fibros.* **22**, 560–563 (2023). 36400713 10.1016/j.jcf.2022.10.010

[15] Dabrowski, M., Bukowy-Bieryllo, Z. & Zietkiewicz, E. Translational readthrough potential of natural termination codons in eucaryotes—the impact of RNA sequence. *RNA Biol.* **12**, 950–958 (2015). 26176195 10.1080/15476286.2015.1068497 PMC4615788

[16] Bidou, L. et al. 2-Guanidino-quinazoline promotes the readthrough of nonsense mutations underlying human genetic diseases. *Proc. Natl Acad. Sci. USA* **119**, e2122004119 (2022). 35994666 10.1073/pnas.2122004119 PMC9436315

[17] Cancer Genome Atlas Research Network, et al. The Cancer Genome Atlas Pan-Cancer analysis project. *Nat. Genet.* **45**, 1113–1120 (2013). 24071849 10.1038/ng.2764 PMC3919969

[18] Cheng, D. T. et al. Memorial Sloan Kettering-Integrated Mutation Profiling of Actionable Cancer Targets (MSK-IMPACT): a hybridization capture-based next-generation sequencing clinical assay for solid tumor molecular oncology. *J. Mol. Diagn.* **17**, 251–264 (2015). 25801821 10.1016/j.jmoldx.2014.12.006 PMC5808190

[19] Matreyek, K. A., Stephany, J. J., Chiasson, M. A., Hasle, N. & Fowler, D. M. An improved platform for functional assessment of large protein libraries in mammalian cells. *Nucleic Acids Res.* **48**, e1 (2020). 31612958 10.1093/nar/gkz910 PMC7145622

[20] Pranke, I. et al. Factors influencing readthrough therapy for frequent cystic fibrosis premature termination codons. *ERJ Open Res.* **4**, 00080-02017 (2018). 29497617 10.1183/23120541.00080-2017 PMC5827411

[21] Luna, S. et al. A global analysis of the reconstitution of PTEN function by translational readthrough of PTEN pathogenic premature termination codons. *Hum. Mutat.* **42**, 551–566 (2021). 33600059 10.1002/humu.24186

[22] Floquet, C., Deforges, J., Rousset, J.-P. & Bidou, L. Rescue of non-sense mutated p53 tumor suppressor gene by aminoglycosides. *Nucleic Acids Res.* **39**, 3350–3362 (2011). 21149266 10.1093/nar/gkq1277 PMC3082906

[23] Loughran, G. et al. Evidence of efficient stop codon readthrough in four mammalian genes. *Nucleic Acids Res.* **42**, 8928–8938 (2014). 25013167 10.1093/nar/gku608 PMC4132726

[24] Green, L. & Goff, S. P. Translational readthrough-promoting drugs enhance pseudoknot-mediated suppression of the stop codon at the Moloney murine leukemia virus gag–pol junction. *J. Gen. Virol.* **96**, 3411–3421 (2015). 26382736 10.1099/jgv.0.000284 PMC5972331

[25] Lee, R. E. et al. Small-molecule eRF3a degraders rescue CFTR nonsense mutations by promoting premature termination codon readthrough. *J. Clin. Invest.* **132**, e154571 (2022). 35900863 10.1172/JCI154571 PMC9479597

[26] Baradaran-Heravi, A. et al. Effect of small molecule eRF3 degraders on premature termination codon readthrough. *Nucleic Acids Res.* **49**, 3692–3708 (2021). 33764477 10.1093/nar/gkab194 PMC8053119

[27] Friesen, W. J. et al. The nucleoside analog clitocine is a potent and efficacious readthrough agent. *RNA* **23**, 567–577 (2017). 28096517 10.1261/rna.060236.116 PMC5340919

[28] Howard, M., Frizzell, R. A. & Bedwell, D. M. Aminoglycoside antibiotics restore CFTR function by overcoming premature stop mutations. *Nat. Med.* **2**, 467–469 (1996). 8597960 10.1038/nm0496-467

[29] Bedwell, D. M. et al. Suppression of a CFTR premature stop mutation in a bronchial epithelial cell line. *Nat. Med.* **3**, 1280–1284 (1997). 9359706 10.1038/nm1197-1280

[30] Sharma, J. et al. A small molecule that induces translational readthrough of CFTR nonsense mutations by eRF1 depletion. *Nat. Commun.* **12**, 4358 (2021). 34272367 10.1038/s41467-021-24575-x PMC8285393

[31] 2024 A molecular glue degrader of eRF1 on the ribosome *Nat. Chem. Biol*. 20 810 811. 10.1038/s41589-023-01522-z 38287153

[32] Cridge, A. G., Crowe-McAuliffe, C., Mathew, S. F. & Tate, W. P. Eukaryotic translational termination efficiency is influenced by the 3′ nucleotides within the ribosomal mRNA channel. *Nucleic Acids Res.* **46**, 1927–1944 (2018). 29325104 10.1093/nar/gkx1315 PMC5829715

[33] Wangen, J. R. & Green, R. Stop codon context influences genome-wide stimulation of termination codon readthrough by aminoglycosides. *eLife* **9**, e52611 (2020). 31971508 10.7554/eLife.52611 PMC7089771

[34] Namy, O., Hatin, I. & Rousset, J. P. Impact of the six nucleotides downstream of the stop codon on translation termination. *EMBO Rep.* **2**, 787–793 (2001). 11520858 10.1093/embo-reports/kve176 PMC1084031

[35] Mottagui-Tabar, S. & Isaksson, L. A. Only the last amino acids in the nascent peptide influence translation termination in *Escherichia coli* genes. *FEBS Lett.* **414**, 165–170 (1997). 9305752 10.1016/S0014-5793(97)00978-2

[36] Mottagui-Tabar, S., Tuite, M. F. & Isaksson, L. A. The influence of 5′ codon context on translation termination in *Saccharomyces cerevisiae*. *Eur. J. Biochem.* **257**, 249–254 (1998). 9799126 10.1046/j.1432-1327.1998.2570249.x

[37] Tork, S., Hatin, I., Rousset, J.-P. & Fabret, C. The major 5′ determinant in stop codon read-through involves two adjacent adenines. *Nucleic Acids Res.* **32**, 415–421 (2004). 14736996 10.1093/nar/gkh201 PMC373328

[38] Cassan, M. & Rousset, J. P. UAG readthrough in mammalian cells: effect of upstream and downstream stop codon contexts reveal different signals. *BMC Mol. Biol.* **2**, 3 (2001). 11242562 10.1186/1471-2199-2-3 PMC29092

[39] Arkov, A. L., Korolev, S. V. & Kisselev, L. L. 5′ contexts of *Escherichia coli* and human termination codons are similar. *Nucleic Acids Res.* **23**, 4712–4716 (1995). 8524665 10.1093/nar/23.22.4712 PMC307448

[40] Sharp, P. M. & Li, W. H. The codon adaptation index—a measure of directional synonymous codon usage bias, and its potential applications. *Nucleic Acids Res.* **15**, 1281–1295 (1987). 3547335 10.1093/nar/15.3.1281 PMC340524

[41] dos Reis, M., Wernisch, L. & Savva, R. Unexpected correlations between gene expression and codon usage bias from microarray data for the whole *Escherichia coli* K-12 genome. *Nucleic Acids Res.* **31**, 6976–6985 (2003). 14627830 10.1093/nar/gkg897 PMC290265

[42] dos Reis, M., Savva, R. & Wernisch, L. Solving the riddle of codon usage preferences: a test for translational selection. *Nucleic Acids Res.* **32**, 5036–5044 (2004). 15448185 10.1093/nar/gkh834 PMC521650

[43] Du, L. et al. Nonaminoglycoside compounds induce readthrough of nonsense mutations. *J. Exp. Med.* **206**, 2285–2297 (2009). 19770270 10.1084/jem.20081940 PMC2757881

[44] Gilad, S. et al. Genotype–phenotype relationships in ataxia-telangiectasia and variants. *Am. J. Hum. Genet.* **62**, 551–561 (1998). 9497252 10.1086/301755 PMC1376949

[45] Spelier, S., van Doorn, E. P. M., van der Ent, C. K., Beekman, J. M. & Koppens, M. A. J. Readthrough compounds for nonsense mutations: bridging the translational gap. *Trends Mol. Med.* **29**, 297–314 (2023). 36828712 10.1016/j.molmed.2023.01.004

[46] Sermet-Gaudelus, I. et al. Ataluren (PTC124) induces cystic fibrosis transmembrane conductance regulator protein expression and activity in children with nonsense mutation cystic fibrosis. *Am. J. Respir. Crit. Care Med.* **182**, 1262–1272 (2010). 20622033 10.1164/rccm.201001-0137OC

[47] Mosallaei, D. et al. Molecular and clinical outcomes after intravenous gentamicin treatment for patients with junctional epidermolysis bullosa caused by nonsense variants. *JAMA Dermatol.* **158**, 366–374 (2022). 35234826 10.1001/jamadermatol.2021.5992 PMC8892363

[48] Finkel, R. S. et al. Phase 2a study of ataluren-mediated dystrophin production in patients with nonsense mutation Duchenne muscular dystrophy. *PLoS ONE* **8**, e81302 (2013). 24349052 10.1371/journal.pone.0081302 PMC3859499

[49] Li, S. et al. Pharmaceuticals promoting premature termination codon readthrough: progress in development. *Biomolecules* **13**, 988 (2023). 37371567 10.3390/biom13060988 PMC10296333

[50] Wu, C., Roy, B., He, F., Yan, K. & Jacobson, A. Poly(A)-binding protein regulates the efficiency of translation termination. *Cell Rep.* **33**, 108399 (2020). 33207198 10.1016/j.celrep.2020.108399 PMC7717110

[51] Müller, M. B. D., Kasturi, P., Jayaraj, G. G. & Hartl, F. U. Mechanisms of readthrough mitigation reveal principles of GCN1-mediated translational quality control. *Cell* **186**, 3227–3244 (2023). 37339632 10.1016/j.cell.2023.05.035 PMC10364623

[52] Sathyan, A., Weinberg, A. I. & Cohen, K. Interpretable AI for bio-medical applications. *Complex Eng. Syst.* **2**, 18 (2022). 37025127 10.20517/ces.2022.41 PMC10074303

[53] Peterman, N. & Levine, E. Sort-seq under the hood: implications of design choices on large-scale characterization of sequence-function relations. *BMC Genomics* **17**, 206 (2016). 26956374 10.1186/s12864-016-2533-5 PMC4784318

[54] Yoon, J., Chung, Y.-J. & Lee, M. STADIUM: species-specific tRNA adaptive index compendium. *Genomics Inform.* **16**, e28 (2018). 30602089 10.5808/GI.2018.16.4.e28 PMC6440659

[55] Alexaki, A. et al. Codon and codon-pair usage tables (CoCoPUTs): facilitating genetic variation analyses and recombinant gene design. *J. Mol. Biol.* **431**, 2434–2441 (2019). 31029701 10.1016/j.jmb.2019.04.021

[56] lehner-lab/Stop_codon_readthrough: v1.0. *Zenodo* zenodo.org/records/12698349 (2024).

