# Supplementary material for: Genome-scale quantification and prediction of pathogenic stop codon readthrough by small molecules
Source: Nat Genet. 2024 Aug 22;56(9):1914–24. doi: 10.1038/s41588-024-01878-5 (PMC11387191; doi:10.1038/s41588-024-01878-5)
Supplement: Supplementary file 1 — Supplementary Notes 1–8 and Supplementary Fig. 1. [file 41588_2024_1878_MOESM1_ESM.pdf]

nature genetics

Supplementary information https://doi.org/10.1038/s41588-024-01878-5

Genome-scale quantification and prediction of pathogenic stop codon readthrough by small molecules

In the format provided by the authors and unedited

Supplementary Note 1: Upper bound on sort-seq readthrough estimations.

Validating the DMS readthrough values for 15 variants, under SRI treatment, using single variant genome integration by flow cytometry (See single variants validation Methods section) allowed us to assess the performance of our DMS sort-seq readthrough assay (Fig. 1d). Correlation value (r=0.95, Pearson) is very high when removing the outlier (correlation on 14/15 variants). The inclusion of this outlier drops the correlation to 0.8. Ten more variants spanning readthrough values around the outlier were individually tested, confirming the loss of accuracy in the upper readthrough range (Extended Data Fig. 1h).

The disagreement in readthrough values between sort-seq and validation in these high readthrough variants responds to a well-known sort-seq related problem[1]. Variants with readthrough distributions smaller than the sorting gate width are more likely to be miscalled. Our gates were designed in the logarithmic scale, meaning that high-readthrough gates have larger widths than low-readthrough gates. This holds especially true for the highest gate, which was designed to include all cells above the top-edge of the second-highest gate, in order to not lose any high-readthrough cells. In turn, only very high-readthrough variants suffer from the problem described above and this results in our assay having a 6% readthrough upper bound (under SRI treatment), preventing us from quantifying the exact readthrough efficiency of variants with readthrough>6% (Fig. 1d). Note that we are underestimating the readthrough of these variants, being the real readthrough equal or higher (but not lower) than our DMS estimate. For how many variants readthrough is underestimated? Using the DMS vs individual measurements data, we detected that DMS readthrough estimates start to saturate for variants with >90% reads in the last gate. We computed the percentage of variants in each treatment that had more than 90% of reads in the highest gate. This number is around 1% (0.41, 0.22, 1.54, 1.73, 1.04, 0.54, 0.57 and 1.31; for CC90009, clitocine, DAP, FUr, G418, gentamicin, SJ6986 and SRI, respectively), meaning that 99% of the library lays on the optimal dynamic range of the assay. We used the average readthrough of these groups of variants as the assay upper limit for each of the treatments, being readthrough of 2.6%, 2.8%, 4.5%, 1.5%, 3.2%, 1.2%, 6.3% and 5.8%; for CC90009, clitocine, DAP, FUr, G418, gentamicin, SJ6986 and SRI, respectively. Same calculations were performed for the NTC library to define the upper limit for each drug condition, with 0.75%, 0.25%, 0.6%, 0.31% and 0.98% variants with >90% reads in the last bin and readthrough values saturating at 2.6%, 2.9%, 3%, 5.1% and 3.6% for clitocine, DAP, G418, SJ6986 and SRI; respectively.

Supplementary Note 2: Sequence effects are preserved across drug concentrations

The optimal drug concentration might be different in other assays and clinical applications. We therefore measured the library under two more SJ6986 concentrations (0.5μM and 20μM) to test the concentration-specificity of the sequence effects. High-quality data (interreplicate correlations r=0.92 and r=0.91, for 0.5μM and 20μM; respectively) showed a shift in the readthrough distribution of the library compared to the 5μM conditions (mean readthrough across all variants is 0.93%, 1.64% and 1.81% for 0.5μM, 5μM and 20μM conditions; respectively), and very good correlations for all three pairwise comparisons, indicating the absence of interaction effects between drug concentration and sequence context (Extended Data Fig.2k,l). 5μM and 20μM readthrough efficiencies show excellent linear correlations (r=0.92), whereas 0.5μM shows a slightly decreased linear correlation (r=0.88 with 5μM and 20μM). This is indicative of a non-linear trend where, in the 5μM and 20μM conditions, very high readthrough variants display lower readthrough than predicted by a linear model (Extended Data Fig.2l). The readthrough of variants with very high readthrough at 0.5μM increases less than expected by a linear model upon 5μM and 20μM treatment. In summary, variants are equally ranked across drug concentrations but their readthrough efficiencies are not always linearly scaled.

Supplementary Note 3: From readthrough signatures to mechanism of action (MOA)

Some of the sequence preferences of particular drugs can be understood from their MOAs. For example, DAP interferes with cytosine 34 modification in tRNATrp and makes it more prone to near-cognate codon pairing[2]. The only near-cognate stop codon to tryptophan (UGG) is UGA, which is consistent with DAP showing the highest UGA-specificity amongst all drugs tested. As another example, in our data the adenosine analog clitocine induces readthrough over UAA and UGA variants. The insertion of clitocine at the wobble position 3 of the codon increases near-cognate pairing, whereas position 2 is intolerant to mispairing[3] potentially preventing clitocine-induced readthrough over UAG variants. Future research might consider the sequence-drug specificities described here to gain additional insights into drug MOAs.

Supplementary Note 4: Comparison of sequence context effects

Sequence downstream of the PTC:

For all drugs, the preferred +1 nucleotide is C. But the rest of the nucleotides show distinct preferences across drugs. For example, U is the most readthrough insensitive +1 nucleotide for SRI and SJ6986, whereas it is the second most sensitive for G418 and gentamicin. Interestingly, drugs with a similar preference for particular stop codons can differ in their preference for the +1 nt. G418 and SRI both promote readthrough of UGA codons over UAG and UAA, but G418 preferences at +1nt are C>U>A>G and SRI's are C>A>G>U (all comparisons adjusted p<1e-05, Wilcoxon). Drugs also show quantitative differences in their +1 nt preferences. For example, readthrough by all drugs is greater for C (n=542) than G (n=1074) at the +1 nt but this preference is stronger for SRI, SJ6986 and G418 (3-, 2.5- and 2.8-fold, respectively) than for clitocine and DAP (1.2- and 1.5-fold, respectively) (adjusted p<2e-16) (Fig.2e, Extended Data Fig.2d).

Readthrough is also modulated by the +2 and +3 nts, and the effects also differ across drugs (Fig.2f, Extended Data Fig.2a). For example, readthrough by SRI is stronger for the downstream nts CAA (n=49) than for CAC (n=28) (1.5-fold, adjusted p=6e-04) and for ACU (n=40) than for ACA (n=41) (1.4-fold, adjusted p=7e-04). In contrast, readthrough of stops with the downstream sequences ACU or ACA is similar for clitocine (0.96-fold, adjusted p=0.5). CU is the top readthrough-promoting dinucleotide at the +1 and +2 positions for all drugs (CU (n=166) vs all other dinucleotides (n=2558) shows 3.3-fold for SRI, 2.4-fold for SJ6986 and 1.4-fold for DAP; all significant at adjusted p<=2e-16) except for G418, for which the readthrough is similar across all variants with C in position +1 but CA displays slightly higher readthrough than CU (CU (n=166) vs CA (n=188) shows 1.8-fold for SRI and 1.4-fold for SJ6986, but 0.8-fold, for G418; all significant at adjusted p<=1e-08). Note that the effect of the +3 position depends on the nucleotides at +1 and +2 positions. For instance, an A at +3 position is the top readthrough nucleotide for SRI when positions +1 and +2 are AA (n(A)=84, n(C/G/T)=206) (1.2-fold, adjusted p=0.02) or CA (n(A)=49, n(C/G/T)=139) (1.3-fold, adjusted p=0.007), whereas A at +3 is the least readthrough-promoting nucleotide when preceded by AG (n(A)=49, n(C/G/T)=138) (0.6-fold, adjusted p=1e-05).

Stop type and downstream sequence interaction

We identified a stop codon-dependent effect of the downstream nucleotides (Fig.2g, Extended Data Fig.2e), indicating genetic interactions between neighboring nucleotides. In the SRI dataset, for UAA stop codons, at position +1 U>G (1.2-fold, adjusted p=0.001, n(U)=213, n(G)=407) whereas for UAG stop codons it is the opposite and at +1 G>U (1.3-fold, adjusted p=2e-11, n(U)=322, n(G)=515). Also, at position +1 A=~G for UAG stop codons (1.1-fold, adjusted p=5e-03, n(A)=477, n(G)=515), but A>G for UGA stop codons (1.8-fold, adjusted p<2e-16, n(A)=766, n(G)=1074). C at +1 position has more efficient readthrough than the other 3 nucleotides in all 3 stop codon contexts.

Sequence upstream of the PTC:

Clustering sequences in our library by the upstream codon reveals upstream preferences for each of the drugs (p<2e-16, Kruskal Wallis test) (Fig.2h, Extended Data Fig.2b).

Similarly to the downstream sequence, these effects are drug-specific (Extended Data Fig.2b). For instance, under SRI and G418 treatment, variants with GAA (n=83) and GAG (n=78) glutamic acid codons immediately before the stop display similar readthrough (1.1-fold, adjusted p=0.06), whereas when treated with DAP, GAA-variants trigger higher readthrough

than GAG-variants (1.5-fold, adjusted p=2e-10) (Fig.2i, Extended Data Fig.2g). Similarly, CGC (n=25) is more sensitive than CGG (n=20) in clitocine (1.7-fold, adjusted p=4e-06) but they undergo similar readthrough under G418 treatment (1.2-fold, adjusted p=0.1) (Extended Data Fig.2h). These examples show that readthrough differs for different codons encoding the same amino acid. For instance, SRI-induced readthrough also differs for others codons encoding arginine (CGC>AGG, 2-fold, adjusted p=2e-05, n(CGC)=25, n(AGG)=33, Extended Data Fig.2h), alanine (GCA>GCU, 1.9-fold, adjusted p=1e-08, n(GCA)=47, n(GCU)=46), valine (GUC>GUA, 1.5-fold, adjusted p=1e-04, n(GUC)=53, n(GUA)=38), and leucine (UUA>CUU, 1.7-fold, n(UUA)=30, n(CUU)=77, adjusted p=1e-07) (Fig.2h).

Supplementary Note 5: Patient response vs readthrough predictions

NCT00458341 and NCT00264888 clinical trials have patient data available, which allowed us to correlate our G418 and SJ6986 readthrough efficiencies with the patient's response to ataluren treatment (ataluren has a mechanism of action that resembles that of aminoglycosides and eRF1 inhibitors[4,5]). In 3/4 cases we observe a suggestive but non-significant correlation (Supplementary Fig.1a,b); however further validation is due.

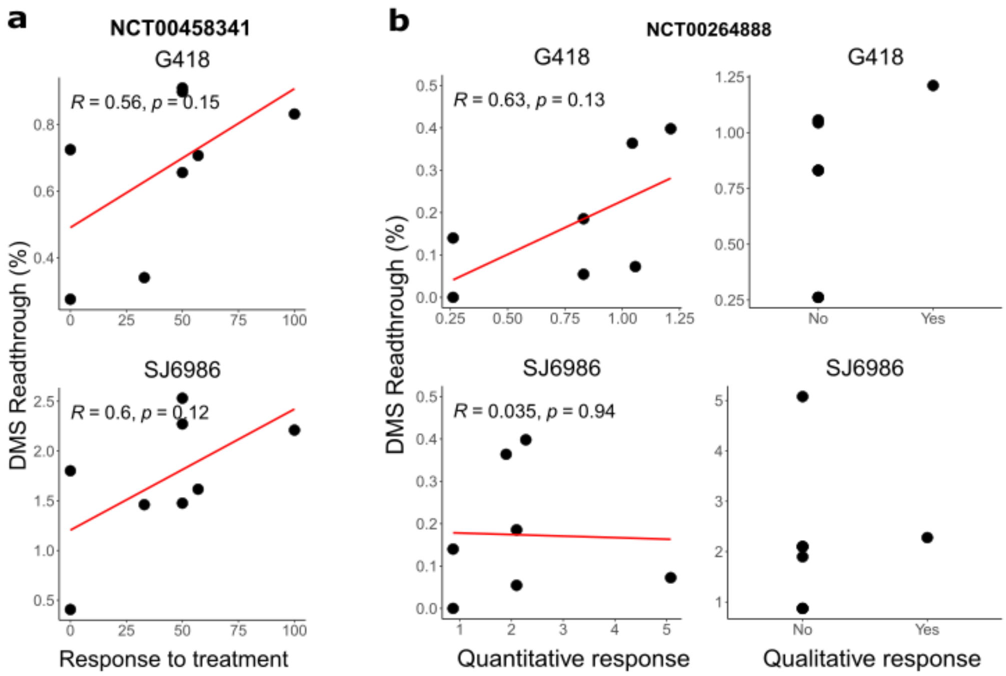

Supplementary Fig.1. Correlation of our G418 and SJ6986 DMS measurements with the response to Ataluren treatment of patients from two different clinical trials, based on mutation identity. Patients in clinical trial NCT00264888 were evaluated using two metrics (named quantitative and qualitative). Pearson's correlation and corresponding *P* values are shown.

Supplementary Note 6: Model design

We computed the Pearson-correlations (continuous features) or chi-squared (Kruskal-Wallis) values (discrete features) between the listed features and readthrough in our dataset (Extended Data Fig. 1f). We generated models by manually including and excluding the top-correlated features. Along this process, we realized that predicted and observed readthrough were non-linearly related. This non linearity issue was fixed when using generalized linear models with logit as the link function. In this scenario, we devised a new term referred to as 'readthrough potential'. Mathematically, it corresponds to the exponential term in the denominator of the model (*Formula 4*). Conceptually, it can be understood as an energetic readthrough value and features additively combine to determine it. The readthrough potential is non-linearly (sigmoid) correlated with the observed/phenotypic readthrough. This is captured in the model by the addition of a sigmoid as a link function between readthrough and readthrough potential.

Models were trained using 90% of the dataset and tested in the remaining 10% of variants (held-out dataset). This process was repeated 10 times, and the mean r^2 across these cross validation rounds was used as the model performance metric. We sought for those features combinations that maximized model performance (r^2) without overfitting it (tested by cross-validation).

Our best performing model (Fig. 4a) used the stop type, the three nucleotides downstream of the UGA (down_123nt), the three nucleotides upstream of the UGA (up_123nt) and the interaction between down_123nt and stop_type as predictor variables (Formula 4, Supplementary Note 8). This was the top-performing model across all drugs.

To compare this model to alternative approaches we conducted four analyses: :

- Run ElasticNet regularized regression with all features in Fig. 2d (Supplementary Table 4) allowing for second order interactions. Model performance was not improved for clitocine and slightly improved for the rest of the drugs (r^2 increased by 1% in CC90009, 1% in DAP, 2% in G418, 3% in SRI and 4% in SJ6986) (Extended Data Fig. 4c).
- Manually test if the inclusion of the stop_type*up_123nt interaction term improved model performance. It did not improve the r^2 in contrast to the inclusion of the stop_type*down_123nt, which increased r^2s by 1-3% over the model without interaction terms (Extended Data Fig. 4d).
- We tested whether a simplified encoding of the +/-3nts sequence maintained the high model performance (Extended Data Fig. 4e). We broke down the up_123nts and down_123nts into three terms each: up1, up2, up3 and down1, down2, down3 terms; reducing complexity of the model by cutting down the number of levels from 64 to 12. The other model terms were not modified, and the interaction between stop type and each of the down1, down2, down3 was included. The simplified models consistently show a decreased performance across drugs, suggesting that the interactions between the nucleotides positions are significantly contributing to readthrough and validating our initial model as the best model.
- To ensure that we were not missing effects of nucleotide positions further downstream of the stop, we designed models including each of the eight nucleotide positions downstream of the PTC independently, and checked the contribution of each term in the model as increase in r^2 (Fig. 4b) by 10 rounds of cross validation (Formula 5, Supplementary Note 8).

Across drugs the +1 and +2 nts are the only positions contributing to readthrough variability across the dataset. Note that the +3 position is not significant in these models, but it slightly but consistently increases model performance when allowing the interactions with the +1 and +2 positions, suggesting that the effect of +3 position is highly dependent on the nucleotide in +1 and +2 positions (down_123nt consistently improved model r^2 1-2% over down_12nts across drugs, data not shown). Importantly, it's likely that further positions also contribute to readthrough but due to our limited library size and design we can't neither accurately nor significantly capture the small effects of such positions.

- Lastly, we also tested the effects of the +4 nucleotides downstream considering all possible interactions. We swapped the down_123nt term for the down_1234nt. r^2 after 10 cross validation rounds was not improved for any of the drugs (data not shown).

We used the same model formulation for the NTC library. The predictions and coefficients of the models can be accessed in Supplementary Tables 12 and 13.

Models for gentamicin, FUr and untreated conditions:

Models trained to predict readthrough for gentamicin (37 PTCs with >1% readthrough) and FUr (153 PTCs with >1%) showed moderate performance (r^2=0.37 and 0.38, respectively) and models trained to predict readthrough without any drug (17 PTCs >1%) were not predictive (r^2=0.02). Interestingly, the untreated dataset shows high correlation with both gentamicin and FUr datasets (Fig. 2b, Extended Data Fig. 1e). Thus, gentamicin and FUr, which have a low readthrough-inducing capacity, mostly increase readthrough of those variants which already undergo some basal readthrough (Extended Data Fig. 1e). Downsampling the number of positive examples for the other drugs suggests that this moderate and poor performance likely reflects the small number of positive examples and that improved performance is likely to be obtained by testing a larger set of PTCs (Extended Data Fig. 4b). For these 3 conditions, predictive models can not be trusted, but note the relevance of the high-confidence measurements for the ~6000 variants of the library in gentamicin, FUr and untreated conditions.

Simplified version of the models without the stop_type*down_123nts interaction term

For the sake of coefficient interpretability we also ran the models without the interaction term stop_type*down_123nts, which incurs only a small decrease of r^2, ranging between 1-3% (Fig. 4d). Cross-validation was used to obtain the mean, standard deviation and significance of the coefficient estimates (Extended Data Fig. 4g-i, Supplementary Table 9, Supplementary Table 13). Note that coefficients are not in the readthrough percentage scale but in the logit space. Importantly, coefficient sizes must be compared across drugs but within each feature.

Pan drug model

We generated a pan drug model by including new terms that would capture the treatment effects on readthrough (combining the data for the six drugs that gave readthrough (>1%) for >3% PTCs, namely clitocine, CC90009, DAP, G418, SJ6986 and SRI) (Formula 6, Supplementary Note 8).. First, a *drug* term was added to the model, which scales the readthrough for each treatment. However, sequence features have different effects depending on the treatment (Fig. 2d-f, Extended Data Fig. 2a-i). To incorporate such effects into the model, we included 4 interaction terms: *drug:stop_type*, *drug:nt_123*, *drug:up_codon* and *drug:nt_123:stop_type* (in the formula below, the *stoptype* term has been abbreviated to *stop)*. Same as before, model performance was assessed for 10 cross validation rounds. Similar to

the drug-specific models, we also run a slightly simplified version of the pan drug model without the stop_type:down_123nt term to ease coefficient interpretability (incurs only a 2% drop in r^2). These coefficients are listed in Supplementary Table 10 and some of them shown in Extended Data Fig. 4j.

Supplementary Note 7: Analyses of predictive models features

To identify features important for model performance, we removed one variable at a time and calculated the drop in cross-validated r2 normalized to the full model r2 (Fig.4d, Extended Data Fig.4f). Feature contributions quantitatively differ across readthrough drug models. For DAP and clitocine, stop codon type explains most of the variance (92% drop in r2 upon stop codon type variable removal) with some contribution of the downstream nts (5-10% drop in r2). In contrast, for SRI and SJ6986, the downstream nts are much more important for model performance (66% and 60% drop in r2 upon removal, respectively) and the dependence on stop codon type is lower (35% and 40% drop in r2 upon removal). For G418 there is a very similar r2 reduction upon removing the stop type and downstream nts (50% and 57% drop in r2 upon removal, respectively). The upstream nt sequence is less important in general than the downstream nt sequence, but there is still a substantial drop in performance when removing it for SRI, SJ6986 and G418 (15%, 15% and 21% drop in r2 upon removal, respectively). The interaction between the stop codon type and the downstream nts is less important for model performance, with r2 dropping by 1-6% upon its removal. Analysis of the pan-drug model yields very similar conclusions (Extended Data Fig.4f).

We also compared the model coefficients for each feature (in a simplified model without the interaction term to aid coefficient interpretability, see Methods) (Fig.4e, Extended Data Fig.4g-i, Supplementary Table 9). The correlations of the coefficients for each feature between drugs reflects the known MOA. For example, the coefficients for SRI are highly correlated with those for SJ6986 and CC90009 for all three types of features (upstream nts, downstream nts and stop codon), suggesting these drugs show a similar dependence on sequence context. In contrast, although the stop codon coefficients are perfectly correlated between SRI and G418 (r=1), the upstream nt coefficients are only poorly correlated (r=0.24). This indicates that readthrough by SRI and G418 respond very similarly to stop codon type but very differently to changes in the upstream sequence. In contrast, readthrough by SRI and clitocine respond similarly to changes in the surrounding sequence context (r=0.83 and r=0.64 for upstream and downstream nt coefficients, respectively) but very differently to the stop codon type (r=0.33). The coefficient analysis was replicated for the pan-drug model (Extended Data Fig.4j, Supplementary Table 10).

Supplementary Note 8: Formulas

Formula 1: $mCherry_{var} = \frac{1}{N} \Sigma_{j=1}^{m} \frac{r_j}{R_j} pc_j fv_j$

Formula 2: $RTp_{var} = \frac{mCherry\ var}{mCherry\ no\ nonsense\ variant}$

Formula 3: $RTp_{ind} = \frac{\%(mCherry+/EGFP+)cells * mCherry\ intensity}{(\%(mCherry+/EGFP+)cells * mCherry\ intensity)\ of\ no{-}nonsense\ variant}$

Formula 4: $RT \sim \frac{1}{1 + e\hat{}-(stoptype + down_{123nt} + up_{123nt} + stoptype{:}down_{123nt})}$

Formula 5:

$$RT \sim \frac{1}{1 + e\hat{} - (stoptype + up_{123nt} + down_{1nt} + down_{2nt} + down_{3nt} + down_{4nt} + down_{5nt} + down_{6nt} + down_{7nt} + \ldots}$$ [illegible]

Formula 6:

$$RT \sim \frac{1}{1 + e\hat{} - (stop + down_{123nt} + up_{123nt} + stop{:}down_{123nt} + drug + drug{:}stop + drug{:}down_{123nt} + drug{:}up_{123nt} + drug \ldots}$$ [illegible]

REFERENCES

1. Peterman, N. & Levine, E. Sort-seq under the hood: implications of design choices on large-scale characterization of sequence-function relations. *BMC Genomics* **17**, 206 (2016).
2. Trzaska, C. *et al.* 2,6-Diaminopurine as a highly potent corrector of UGA nonsense mutations. *Nat. Commun.* **11**, 1509 (2020).
3. Roy, B., Leszyk, J. D., Mangus, D. A. & Jacobson, A. Nonsense suppression by near-cognate tRNAs employs alternative base pairing at codon positions 1 and 3. *Proc. Natl. Acad. Sci. U. S. A.* **112**, 3038–3043 (2015).
4. Ng, M. Y., Li, H., Ghelfi, M. D., Goldman, Y. E. & Cooperman, B. S. Ataluren and aminoglycosides stimulate read-through of nonsense codons by orthogonal mechanisms. *Proc. Natl. Acad. Sci. U. S. A.* **118**, (2021).
5. Huang, S. *et al.* Ataluren binds to multiple protein synthesis apparatus sites and competitively inhibits release factor-dependent termination. *Nat. Commun.* **13**, 2413 (2022).
