# Supplementary material for: Genome-scale quantification and prediction of pathogenic stop codon readthrough by small molecules
Source: Nat Genet. 2024 Aug 22;56(9):1914–24. doi: 10.1038/s41588-024-01878-5 (PMC11387191; doi:10.1038/s41588-024-01878-5)
Supplement: Supplementary file 2 — Reporting Summary [file 41588_2024_1878_MOESM2_ESM.pdf]

Corresponding author(s): Fran Supek, Ben Lehner

Last updated by author(s): 14/6/24

Reporting Summary

Nature Portfolio wishes to improve the reproducibility of the work that we publish. This form provides structure for consistency and transparency in reporting. For further information on Nature Portfolio policies, see our Editorial Policies and the Editorial Policy Checklist.

Statistics

For all statistical analyses, confirm that the following items are present in the figure legend, table legend, main text, or Methods section.

n/a	Confirmed	
☐	☒	The exact sample size (n) for each experimental group/condition, given as a discrete number and unit of measurement
☐	☒	A statement on whether measurements were taken from distinct samples or whether the same sample was measured repeatedly
☐	☒	The statistical test(s) used AND whether they are one- or two-sided<br>*Only common tests should be described solely by name; describe more complex techniques in the Methods section.*
☐	☒	A description of all covariates tested
☐	☒	A description of any assumptions or corrections, such as tests of normality and adjustment for multiple comparisons
☐	☒	A full description of the statistical parameters including central tendency (e.g. means) or other basic estimates (e.g. regression coefficient) AND variation (e.g. standard deviation) or associated estimates of uncertainty (e.g. confidence intervals)
☐	☒	For null hypothesis testing, the test statistic (e.g. F, t, r) with confidence intervals, effect sizes, degrees of freedom and P value noted<br>*Give P values as exact values whenever suitable.*
☒	☐	For Bayesian analysis, information on the choice of priors and Markov chain Monte Carlo settings
☒	☐	For hierarchical and complex designs, identification of the appropriate level for tests and full reporting of outcomes
☐	☒	Estimates of effect sizes (e.g. Cohen's d, Pearson's r), indicating how they were calculated

Our web collection on statistics for biologists contains articles on many of the points above.

Software and code

Policy information about availability of computer code

Data collection	FastQ files from paired-end sequencing of all experiments were processed with DiMSum v1.2.9 using default settings with minor adjustments: https://github.com/lehner-lab/DiMSum. Experimental design files and command-line options required for running DiMSum on these datasets are available on GitHub (https://github.com/lehner-lab/Stop_codon_readthrough).
Data analysis	Source code is available at https://github.com/lehner-lab/DiMSum. Source code for all downstream analyses and to reproduce all figures described here is available at https://github.com/lehner-lab/Stop_codon_readthrough and has been archived to Zenodo (https://zenodo.org/records/12698349). FACS data was analyzed with BD FACS (TM) Software (1.0.0.650).

For manuscripts utilizing custom algorithms or software that are central to the research but not yet described in published literature, software must be made available to editors and reviewers. We strongly encourage code deposition in a community repository (e.g. GitHub). See the Nature Portfolio guidelines for submitting code & software for further information.

Data

Policy information about availability of data

All manuscripts must include a data availability statement. This statement should provide the following information, where applicable:

- Accession codes, unique identifiers, or web links for publicly available datasets
- A description of any restrictions on data availability
- For clinical datasets or third party data, please ensure that the statement adheres to our policy

All DNA sequencing data have been deposited in the Sequence Read Archive (SRA) with accession number PRJNA996618: http://www.ncbi.nlm.nih.gov/bioproject/996618 (PTCs) and PRJNA1073909: http://www.ncbi.nlm.nih.gov/bioproject/1073909 (NTCs). The readthrough efficiency predictions have been made available through the Figshare repository at https://figshare.com/articles/dataset/Readthrough_predictions/23708901 and via a digital object identifier (doi:10.6084/m9.figshare.23708901). All readthrough measurements are provided in Extended Data Tables 3, 11.
The MSK-IMPACT and TCGA datasets were downloaded from cBioPortal (https://www.cbioportal.org/) on 02.06.2021. The ClinVar dataset was downloaded from https://ftp.ncbi.nlm.nih.gov/pub/clinvar/vcf_GRCh38/ on 03.06.2021.

Research involving human participants, their data, or biological material

Policy information about studies with human participants or human data. See also policy information about sex, gender (identity/presentation), and sexual orientation and race, ethnicity and racism.

Reporting on sex and gender	*Use the terms sex (biological attribute) and gender (shaped by social and cultural circumstances) carefully in order to avoid confusing both terms. Indicate if findings apply to only one sex or gender; describe whether sex and gender were considered in study design; whether sex and/or gender was determined based on self-reporting or assigned and methods used.<br>Provide in the source data disaggregated sex and gender data, where this information has been collected, and if consent has been obtained for sharing of individual-level data; provide overall numbers in this Reporting Summary. Please state if this information has not been collected.<br>Report sex- and gender-based analyses where performed, justify reasons for lack of sex- and gender-based analysis.*
Reporting on race, ethnicity, or other socially relevant groupings	*Please specify the socially constructed or socially relevant categorization variable(s) used in your manuscript and explain why they were used. Please note that such variables should not be used as proxies for other socially constructed/relevant variables (for example, race or ethnicity should not be used as a proxy for socioeconomic status).<br>Provide clear definitions of the relevant terms used, how they were provided (by the participants/respondents, the researchers, or third parties), and the method(s) used to classify people into the different categories (e.g. self-report, census or administrative data, social media data, etc.)<br>Please provide details about how you controlled for confounding variables in your analyses.*
Population characteristics	*Describe the covariate-relevant population characteristics of the human research participants (e.g. age, genotypic information, past and current diagnosis and treatment categories). If you filled out the behavioural & social sciences study design questions and have nothing to add here, write "See above."*
Recruitment	*Describe how participants were recruited. Outline any potential self-selection bias or other biases that may be present and how these are likely to impact results.*
Ethics oversight	*Identify the organization(s) that approved the study protocol.*

Note that full information on the approval of the study protocol must also be provided in the manuscript.

Field-specific reporting

Please select the one below that is the best fit for your research. If you are not sure, read the appropriate sections before making your selection.

☒ Life sciences ☐ Behavioural & social sciences ☐ Ecological, evolutionary & environmental sciences

For a reference copy of the document with all sections, see nature.com/documents/nr-reporting-summary-flat.pdf

Life sciences study design

All studies must disclose on these points even when the disclosure is negative.

Sample size	5837 variants in PTC library, 18824 in NTC library. During library construction, we used several fold larger number of cells to ensure that each variant was integrated in ~100 cells. For the PTC library, the library size (n=5837) was chosen based on a) to have a good representativity of sequence contexts around the stop (see the same nucleotide-position combination in many different variants to be able to regress out the effects on readthrough) b) could be fit in a Twist Biosciences Oligopool at an affordable price. For the NTCs library, we took the stop codon sequence for all human protein coding genes.
Data exclusions	Sequencing reads that did not pass the QC filters using DiMSum v1.2.9 (https://github.com/lehner-lab/DiMSum) were excluded. For model generation, variants with very low read coverage (<10 reads) were discarded.

Replication	All sort-sequencing experiments were performed in duplicates. All attempts at replication were successful.
Randomization	Randomization strategies are used to randomly allocate patients in two or more groups to control for potentially influencing factors which might affect the study outcome. Since we don't conduct experiments neither with animals nor patients, the randomization strategies do not apply.
Blinding	A blind experiment attempts to withdld information which might influence the participants of the study until the end of the experiment. Our study doesn't comprise patients, so blinding strategies were not applied.

Reporting for specific materials, systems and methods

We require information from authors about some types of materials, experimental systems and methods used in many studies. Here, indicate whether each material, system or method listed is relevant to your study. If you are not sure if a list item applies to your research, read the appropriate section before selecting a response.

Materials & experimental systems

n/a	Involved in the study
☒	☐ Antibodies
☐	☒ Eukaryotic cell lines
☒	☐ Palaeontology and archaeology
☒	☐ Animals and other organisms
☒	☐ Clinical data
☒	☐ Dual use research of concern
☒	☐ Plants

Methods

n/a	Involved in the study
☒	☐ ChIP-seq
☐	☒ Flow cytometry
☒	☐ MRI-based neuroimaging

Eukaryotic cell lines

Policy information about cell lines and Sex and Gender in Research

Cell line source(s)	TetBxB1BFP-iCasp-Blast Clone 12 HEK293T (Matreyek et al., 2020, Nucleic Acid Res; from Douglas M Fowler lab, University of Washington), HeLa (ATCC), MCF7 (ATCC)
Authentication	The cell lines were not authenticated
Mycoplasma contamination	Cell lines were monthly tested for mycoplasma infection. All tests were negative.
Commonly misidentified lines (See ICLAC register)	We didn't use any commonly misidentified cell lines.

Flow Cytometry

Plots

Confirm that:

☒ The axis labels state the marker and fluorochrome used (e.g. CD4-FITC).

☒ The axis scales are clearly visible. Include numbers along axes only for bottom left plot of group (a 'group' is an analysis of identical markers).

☒ All plots are contour plots with outliers or pseudocolor plots.

☒ A numerical value for number of cells or percentage (with statistics) is provided.

Methodology

Sample preparation	TetBxB1BFP-iCasp-Blast Clone 12 HEK293T cells were grown on standard culture plates in DMEM supplemented with 10% FBS tetracycline-free and without antibiotics. They were split before reaching confluency to maintain cell health. Cells were detached with Trypsin, spun down and washed with PBS. For the sort-seq experiments, cells were treated with doxy to induce the expression of the transcript, and after 24hrs the drug was added to the medium for 48h more. We used high volumes of cells to ensure that each variant was represented >100 times in the cell population. Before sorting, the same amount of cells as those sorted in each bin were withdrawn for sequencing as a pre-sorting control.
Instrument	BD Influx (TM) Cell Sorter
Software	BD FACS (TM) Software (1.0.0.650).
Cell population abundance	The percentage of EGFP+/mCherry+ population was very variable across drugs, ranging from 9% (in untreated conditions) up to 80% (in SRI treatment). Also, the percentage of cells in each of the EGFP+/mCherry+ subpopulations (gates) was very

	variable across drugs. Purities were determined by sorting the cells and then resorting each of the gates separately. Purity was quantified as the number of cells that, when resorted, fell within their gate of origin. Purities were very high across treatments (~85-90%), and more specifically were higher in the upper gates than in the lower gates, because the width of the gates is higher for the upper than for the lower gates.
Gating strategy	Cells were gated by forward scattering area and by side scattering area to retain whole cells, forward scattering width and height to separate discard aggregates, and by DAPI-staining to retain only recombined and alive cells. EGFP and mCherry fluorescence were excited with a 488nm and 561nm lasers and recorded with a 530/40 BP and 593/40 BP channels, respectively. EGFP+ cells were sorted based on mCherry expression into four populations. For most of the populations, 400K cells were sorted. However, for some minor populations representing <2% of the total population, we sorted less cells (100-200K)

☒ Tick this box to confirm that a figure exemplifying the gating strategy is provided in the Supplementary Information.
